# The ichthyofauna of a poorly known area in the middle-southern Espinhaço mountain range, state of Minas Gerais, Brazil: diagnostics and identification keys

**DOI:** 10.3897/zookeys.1054.67554

**Published:** 2021-08-03

**Authors:** Sérgio Alexandre dos Santos, Marcelo Ribeiro de Britto

**Affiliations:** 1 Museu Nacional, Universidade Federal do Rio de Janeiro, Departamento de Vertebrados, Quinta da Boa Vista s/n. CEP 20.940-040, Rio de Janeiro, RJ, Brazil Universidade Federal do Rio de Janeiro Rio de Janeiro Brazil

**Keywords:** Headwater, inventory, rio Doce, rio Paraúna, rio Santo Antônio, rio São Francisco, taxonomy

## Abstract

Knowledge about the taxonomy and fish composition from the upper rio Paraúna (rio São Francisco basin) and upper rio Santo Antônio (rio Doce basin) in the middle portion of the Southern Espinhaço mountain range, state of Minas Gerais, Brazil is still incipient. Only few studies focusing on ichthyofaunistic diagnostic and species descriptions in the lower stretches of the rio Santo Antônio are available. Herein the aim was to provide a species list of the freshwater ichthyofauna from the headwaters of both basins in such region, and to verify the occurrence of threatened, exotic, and potentially new species. Sixty species were registered, with 34 associated to the upper rio Paraúna, and 40 to the upper rio Santo Antônio. Two species are included in some threatened category, three are exotics, and 14 represent potentially new species. An identification key of the fish species recorded in the area is also provided.

## Introduction

The Espinhaço mountain range is one of the most diverse areas in Brazil, presenting a poorly known fauna with high degree of endemism and records of new fish species in recent years (Alves et al. 2008). This scene is particularly reinforced in the headwaters of the rio Doce and rio São Francisco basins. This mountain range acts as important watershed divide of three of the main hydrographic systems from the central-south region of the state of Minas Gerais: rio São Francisco, rio Doce, and rio Jequitinhonha basins. A rich and diversified ichthyofauna is found in these basins. Recent studies show an estimated richness of ca. 240 native species in the rio São Francisco basin (Barbosa et al. 2017), 110 native species in the rio Doce basin (Bueno et al. 2021), and numbers varying between ca. 50 to 70 native species in the rio Jequitinhonha basin (Andrade Neto 2010; Bueno et al. 2021).

The complex of mountains in such a region presents a considerable diversity of fishes that, among other reasons, primarily supported the inclusion of such basins in the list of priority areas for fish conservation in the state of Minas Gerais (Drummond et al. 2005). Despite the great importance of the area, the lack of information about the fish taxonomy occurring there can impact attempts for biodiversity conservation. Additionally, there has been an increase of anthropogenic pressure in the region, especially due to large projects, such as mining and hydroelectric power plants (Vieira 2006, 2010; pers. obs. 2011). Such pressures bring out the need for more studies in an attempt to reduce the knowledge gaps about species taxonomy, enabling a better understanding of continental freshwater fish distribution patterns, and proposition of conservation measures (Menezes et al. 2007).

Herein we aim to present a species list of the ichthyofauna from the headwaters of the middle portion of southern Espinhaço mountain range: the upper rio Paraúna (rio São Francisco basin) and the upper rio Santo Antônio (rio Doce basin). In addition, we propose a dichotomous identification key for the fish species found in the region.

## Material and methods

### Study area

**Figure 1.**
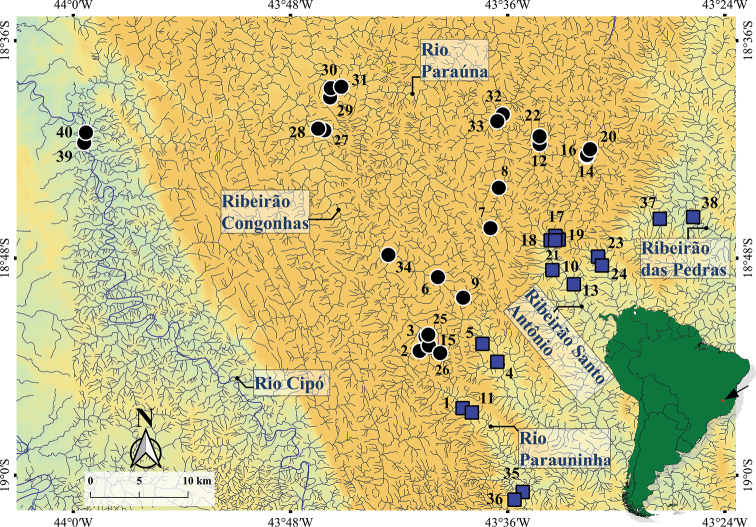
Sample localities in the middle-southern Espinhaço mountain range, Minas Gerais state, Brazil. Numbers matches sample localities in Table 1. Upper rio Paraúna drainages (black circle); upper rio Santo Antônio drainages (blue square); black arrow indicates study area.

The middle-southern Espinhaço mountain range (SEMR) is located in the central-south region of the state of Minas Gerais, Brazil, in an area between the municipalities of Conceição do Mato Dentro, Alvorada de Minas, Congonhas do Norte, Presidente Kubitschek, and Santana de Pirapama. The rio Paraúna is an affluent of the rio das Velhas, and the latter is one of the most important tributaries of the rio São Francisco on its right bank. In its turn, the rio Santo Antônio is one of the most important tributaries of the rio Doce basin on its left bank. Throughout these drainages we sampled 40 localities, which drain the watershed of the upper rio Paraúna and upper rio Santo Antônio (Table 1; Figs 1, 2).

**Figure 2.**
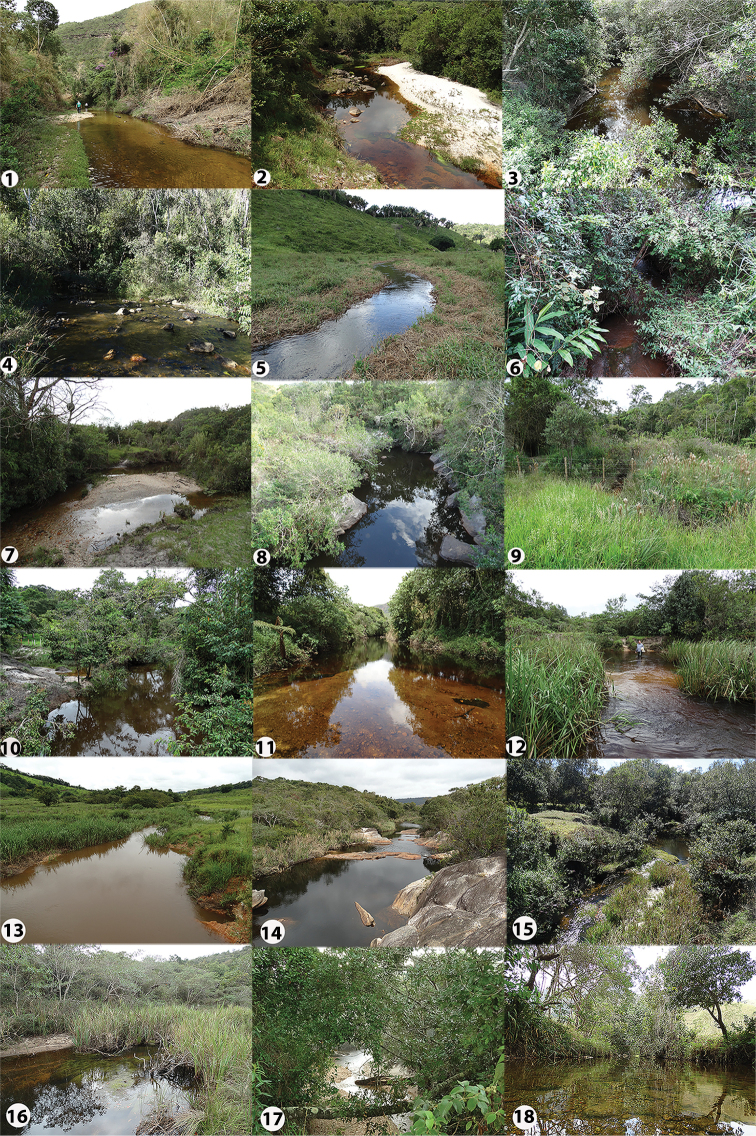
Sample localities in the middle-southern Espinhaço mountain range, Minas Gerais state, Brazil. Numbers follow Figure 1 and Table 1.

**Figure 2.**
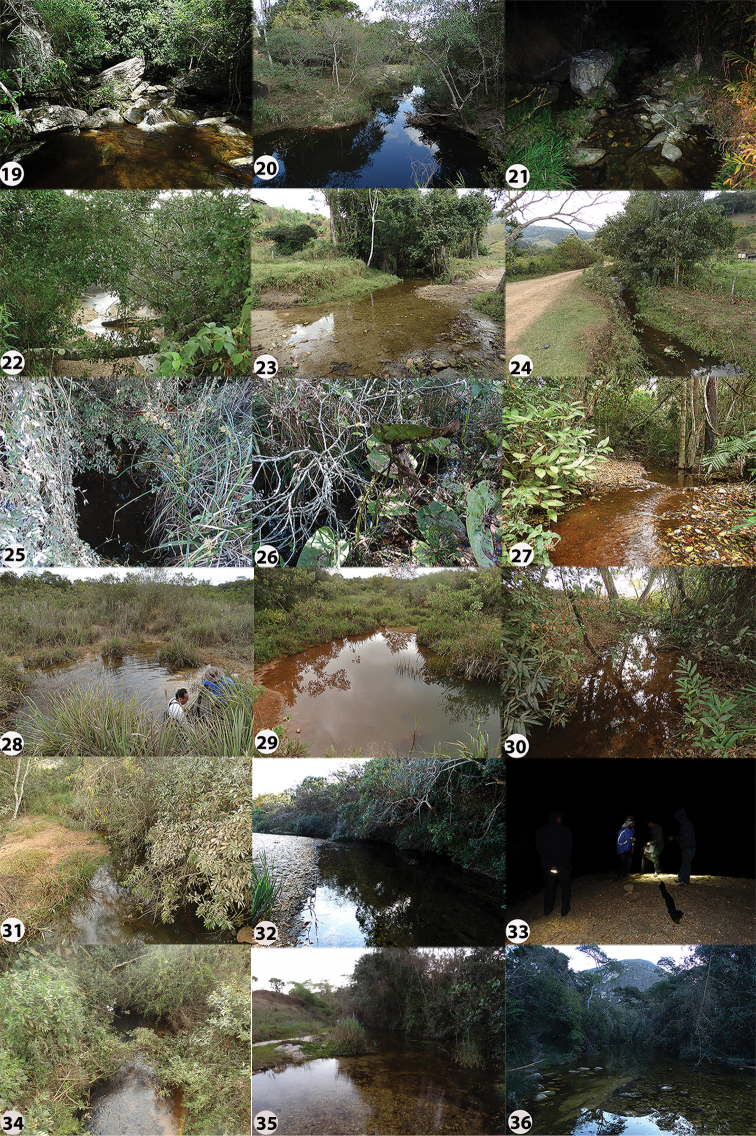
Continued.

**Table 1.** Sampled localities in the middle-southern Espinhaço mountain range, hydrographic basins, and coordinates.

Site	Locality	Drainage	Basin	Coordinates
1	Rio Parauninha	Santo Antônio	Doce	18°56'16"S; 43°38'29"W
2	Ribeirão Congonhas	Rio das Velhas	São Francisco	18°53'07"S; 43°40'52"W
3	Ribeirão Congonhas	Rio das Velhas	São Francisco	18°52'20"S; 43°40'33"W
4	Rio Lambari ou Cachoeira do Jacu	Santo Antônio	Doce	18°53'43"S; 43°36'34"W
5	Rio Lambari ou Cachoeira do Jacu	Santo Antônio	Doce	18°52'43"S; 43°37'24"W
6	Córrego Santa Maria	Rio das Velhas	São Francisco	18°49'03"S; 43°39'51"W
7	Ribeirão Gurutuba	Rio das Velhas	São Francisco	18°46'20"S; 43°36'57"W
8	Ribeirão Gurutuba	Rio das Velhas	São Francisco	18°44'07"S; 43°36'29"W
9	Córrego Santa Maria	Rio das Velhas	São Francisco	18°50'11"S; 43°38'28"W
10	Ribeirão Santo Antônio ou Cruzeiro	Santo Antônio	Doce	18°48'39"S; 43°33'32"W
11	Rio Parauninha	Santo Antônio	Doce	18°56'31"S; 43°37'59"W
12	Rio Paraúna	Rio das Velhas	São Francisco	18°41'42"S; 43°34'14"W
13	Ribeirão Santo Antônio ou Cruzeiro	Santo Antônio	Doce	18°49'26"S; 43°32'21"W
14	Rio Paraúna	Rio das Velhas	São Francisco	18°42'23"S; 43°31'38"W
15	Ribeirão Congonhas	Rio das Velhas	São Francisco	18°52'49"S; 43°40'21"W
16	Córrego Ponte Nova	Rio das Velhas	São Francisco	18°42'18"S; 43°31'37"W
17	Córrego sem nome	Santo Antônio	Doce	18°46'46"S; 43°33'22"W
18	Ribeirão Santo Antônio ou Cruzeiro	Santo Antônio	Doce	18°47'01"S; 43°33'38"W
19	Córrego Pica-pau	Santo Antônio	Doce	18°46'58"S; 43°33'11"W
20	Córrego Ponte Nova	Rio das Velhas	São Francisco	18°41'59"S; 43°31'27"W
21	Córrego Pica-pau	Santo Antônio	Doce	18°47'01"S; 43°33'23"W
22	Córrego sem nome	Rio das Velhas	São Francisco	18°41'16"S; 43°34'14"W
23	Ribeirão Santo Antônio do Norte	Santo Antônio	Doce	18°47'55"S; 43°31'01"W
24	Ribeirão Santo Antônio do Norte	Santo Antônio	Doce	18°48'24"S; 43°30'47"W
25	Córrego sem nome	Rio das Velhas	São Francisco	18°52'13"S; 43°40'23"W
26	Córrego sem nome	Rio das Velhas	São Francisco	18°53'14"S; 43°39'44"W
27	Córrego dos Esteios	Rio das Velhas	São Francisco	18°40'57"S; 43°46'08"W
28	Córrego dos Esteios	Rio das Velhas	São Francisco	18°40'51"S; 43°46'28"W
29	Córrego sem nome	Rio das Velhas	São Francisco	18°39'08"S; 43°45'49"W
30	Córrego sem nome	Rio das Velhas	São Francisco	18°38'37"S; 43°45'46"W
31	Córrego do Sítio	Rio das Velhas	São Francisco	18°38'33"S; 43°45'11"W
32	Ribeirão do Tijucal	Rio das Velhas	São Francisco	18°40'04"S; 43°36'16"W
33	Ribeirão do Tijucal	Rio das Velhas	São Francisco	18°40'27"S; 43°36'35"W
34	Córrego Santa Maria	Rio das Velhas	São Francisco	18°47'49"S; 43°42'35"W
35	Córrego Capão	Santo Antônio	Doce	19° 00'55"S; 43°35'10"W
36	Córrego Capão	Santo Antônio	Doce	19° 01'20"S; 43°35'37"W
37	Ribeirão das Pedras	Santo Antônio	Doce	18°45'50"S; 43°27'36"W
38	Ribeirão das Pedras	Santo Antônio	Doce	18°45'44"S; 43°25'45"W
39	Calha principal do Rio Cipó	Rio das Velhas	São Francisco	18°41'38"S; 43°59'24"W
40	Córrego sem nome	Rio das Velhas	São Francisco	18°41'04"S; 43°59'18"W

### Ichthyofaunistic sampling

Two field expeditions were carried out in March and July-August 2016, under collecting permits 8142-1 and 52362-1, issued by the Instituto Chico Mendes de Conservação da Biodiversidade and 041-2016, by the Instituto Estadual de Florestas (IEF-MG). For this purpose, fishing artifacts commonly employed in ichthyological studies were used, which included aluminum ring sieves and 1 mm-mesh mosquito net, aluminum cord and hoop socks and 1 mm mesh mosquito net, 15 mm- and 20 mm-mesh netting and bamboo rods with nylon line, and worm used as bait. Samples were taken during the day and occasionally in the early evening.

The care and use of experimental animals complied with animal welfare laws, guidelines and policies under Collecting Permit by Instituto Brasileiro do Meio Ambiente e dos Recursos Naturais Renováveis (SISBIO #8142-1). Afterwards, the collected specimens were preserved in 10% formalin solution and transferred to 70% ethanol solution. The sampled material was deposited at the Ichthyological Collection of the Museu Nacional, Universidade Federal do Rio de Janeiro (**MNRJ**, Rio de Janeiro, Brazil). In order to increase recorded species richness and more reliable sampling of actual diversity, specimens available in different scientific collections were analyzed, such as the Museu de Ciências e Tecnologia, Pontifícia Universidade Católica do Rio Grande do Sul (**MCP**, Porto Alegre); Museu de Zoologia da Universidade de São Paulo (**MZUSP**, São Paulo); Museu de Zoologia da Universidade Estadual de Campinas (**ZUEC**, Campinas); Museu de Ciências Naturais da Pontifícia Universidade Católica de Minas Gerais (**MCNIP**, Belo Horizonte); and Naturhistorisches Museum (**NMW**, Wien) (Table 2). Comparative material was also verified in these institutions. Geographical distribution of species was based on Fricke et al. (2021).

**Table 2.** Fish species found in the middle-southern Espinhaço mountain range, Minas Gerais state, Brazil. Legend: (+) presence of species in drainages. CG = Ribeirão Congonhas; CP = Rio Cipó; PA = Rio Paraúna; PD = Ribeirão das Pedras; PH = Rio Parauninha; SA = Rio Santo Antônio; ^1^ – threatened species, according to COPAM (2010) and/or (MMA, 2018); ^2^ – endemic species from Rio São Francisco basin; ^3^ – endemic species from Rio Doce basin; ^4^ – exotic species to Rio São Francisco and/or Rio Doce basin. The order sequence follows Buckup et al. (2007), except for the updates in Bryconidae, Cichliformes and Stethaprioninae (see Material and methods). Genera and species sequences are given in alphabetic order.

Species	Upper Rio Paraúna			Upper Rio Santo Antônio		
	CG	CP	PA	PD	PH	SA
**Order Characiformes**						
**Family Prochilodontidae**						
*Prochiloduscostatus*^2^ Valenciennes, 1850		+				
**Family Anostomidae**						
*Hypomasticusmormyrops* (Steindachner, 1875)				+		
*Hypomasticusthayeri*^1^ (Borodin, 1929)				+		
*Leporellusvittatus* (Valenciennes, 1850)		+				
*Leporinusamblyrhynchus*^4^ Garavello & Britski, 1987		+				
*Leporinuscopelandii* Steindachner, 1875						+
*Leporinusmarcgravii*^2^ Lütken, 1875		+				
*Leporinustaeniatus*^2^ Lütken, 1875		+				
*Megaleporinusobtusidens* (Valenciennes, 1837)		+				
**Family Crenuchidae**						
*Characidiumfasciatum* Reinhardt, 1867			+			
*Characidium* sp. A				+	+	
*Characidium* sp. B				+		+
*Characidium* sp. C				+		+
**Family Bryconidae**						
*Bryconopalinus*^1^ (Cuvier, 1819)				+		
**Family Characidae**						
*Phenacogasterfranciscoensis*^2^ Eigenmann, 1911		+				
*Astyanaxlacustris* Lütken, 1875	+	+	+		+	+
*Astyanax* sp.	+				+	
*Deuterodongiton* Eigenmann, 1908				+	+	+
*Deuterodonintermedius* Eigenmann, 1908				+		
*Deuterodonpedri*^3^ Eigenmann, 1908				+	+	+
Deuterodon aff. taeniatus				+	+	+
*Deuterodon* sp.						+
*Psalidodonrivularis*^2^ (Lütken, 1875)	+		+		+	
*Psalidodon* sp.			+			+
*Hasemanianana*^2^ (Lütken, 1875)	+		+	+		+
*Hasemania* sp.	+		+			
*Knodusmoenkhausii* Eigenmann & Kennedy, 1903				+		+
*Oligosarcusargenteus* Günther, 1864	+			+	+	+
*Piabinaargentea* Reinhardt, 1867		+				
**Family Erythrinidae**						
*Hopliasintermedius* (Günther, 1864)	+	+	+	+	+	+
**Order Siluriformes**						
**Family Aspredinidae**						
*Bunocephalushartti*^2^ Carvalho, Cardoso, Friel & Reis, 2015		+				
**Family Trichomycteridae**						
*Trichomycterusalternatus* (Eigenmann, 1917)			+	+	+	+
*Trichomycterusmelanopygius* Reis, dos Santos, Britto, Volpi & de Pinna, 2020				+	+	+
*Trichomycterus* sp. A				+		
*Trichomycterus* sp. B			+	+	+	+
**Family Callichthyidae**						
*Callichthyscallichthys* (Linnaeus, 1758)	+				+	+
*Hoplosternumlittorale* (Hancock, 1828)			+			
**Family Loricariidae**						
*Euryochusthysanos* Pereira & Reis, 2017				+		
*Neoplecostomus* sp. A				+	+	+
*Neoplecostomus* sp. B			+			
*Harttiaintermontana*^3^ Oliveira & Oyakawa, 2019						+
*Harttia* sp.				+		
*Hypostomusfrancisci* (Lütken, 1874)		+				
*Hypostomus* sp.				+		
*Pareiorhaphisscutula*^3^ Pereira, Vieira & Reis, 2010				+	+	+
*Pareiorhaphisvetula*^3^ Pereira, Lehmann & Reis, 2016					+	+
*Pareiorhaphis* sp.			+			
**Family Heptapteridae**						
*Phenacorhamdiatenebrosa* (Schubart, 1964)		+				
*Rhamdiaquelen* group	+	+	+	+	+	
**Family Pimelodidae**						
*Duopalatinusemarginatus*^2^ (Valenciennes, 1840)		+				
*Pimelodusfur*^2^ (Lütken, 1874)		+				
**Order Gymnotiformes**						
**Family Gymnotidae**						
*Gymnotuscarapo* group	+		+	+	+	+
**Family Sternopygidae**						
*Eigenmanniavirescens* (Valenciennes, 1836)		+				
**Order Cyprinodontiformes**						
**Family Poeciliidae**						
*Phallocerosharpagos* Lucinda, 2008					+	
*Phallocerosuai*^2^ Lucinda, 2008					+	+
*Poeciliareticulata*^4^ Peters 1859	+		+			+
**Order Synbranchiformes**						
**Family Synbranchidae**						
*Synbranchusmarmoratus* group						+
**Order Cichliformes**						
**Family Cichlidae**						
*Australoherosmattosi*^2^ Ottoni, 2012			+			
*Australoheros* sp.					+	
*Geophagusbrasiliensis* (Quoy & Gaimard ,1824)	+		+	+	+	+

### Identification keys

The identification keys are exclusive for identifying species that occur in the upper rio Paraúna (rio São Francisco basin) and upper rio Santo Antônio (rio Doce basin), state of Minas Gerais, Brazil. We first present a dichotomous key based on morphological characters to identify fish orders. Registered orders that have representatives of a single family, have their respective names given in parentheses, including the number of genera and species associated with the family. A second dichotomous key is organized sequentially, following the classification adopted by Buckup et al. (2007), except for some taxonomic updates, such as order Perciformes, which was treated as Cichliformes, following Nelson et al. (2016), subfamily Stethaprioninae (Téran et al. 2020), and family Bryconidae (Fricke et al. 2021). Families which have only one species present in the study area are identified in the second dichotomous key. For those families which have more than one species present, another key is presented (one key per family).

Species were identified through available publications and comparisons with reference material in fish collections. Meristic and morphometric data were taken point to point, whenever possible, on the left side of specimens. Morphometric data were taken using digital calipers under a stereomicroscope. Standard length of the specimens was abbreviated as SL and measurements were taken in millimeters. Whenever necessary, analysis of branchial arch, teeth, procurrent rays of caudal fin, and vertebrae were obtained from cleared and stained material, according to Taylor and Van Dyke (1985). Whenever possible, we also made X-Ray images of some specimens for bone structure analyses – such images were made at the Laboratório de Radiografia from Departamento de Vertebrados, Museu Nacional (Faxitron X-ray, model MX-20 DC12). Osteological terminologies were based on specific bibliographies for each group. Some diagnostic characters shown in the identification key such as number of fin rays and pored scales in lateral line, may present some overlap between different species. However, those characters aim to complement the diagnosis of each species. General distributions of species were based on the published literature for each taxon, and represent their respective ranges of occurrence in the Neotropical region. Distributions of species shown in the identification key were solely based on the records of the study area. Supplementary file 1 summarizes voucher information and comparative material with institutional acronyms, following Sabaj (2019).

## Results

### Material examined

**Parodontidae**: *Apareiodonibitiensis* Amaral Campos, 1944 (n = 3); **Curimatidae**: *Cyphocharaxgilbert* (Quoy & Gaimard, 1824) (n = 1); **Prochilodontidae**: *Prochiloduscostatus* Valenciennes, 1850 (n = 1); **Anostomidae**: *Hypomasticusmormyrops* (Steindachner, 1875) (n = 8); *Hypomasticusthayeri* Borodin, 1929 (n = 24); *Leporellusvittatus* (Valenciennes, 1850) (n = 1); *Leporinusamblyrhynchus* Garavello & Britski, 1987 (n = 1); *Leporinuscopelandii* (Steindachner, 1875) (n = 1); *Leporinusmarcgravii* Lütken, 1875 (n = 1); *Leporinustaeniatus* Lütken, 1875 (n = 2); *Megaleporinusobtusidens* (Valenciennes, 1837) (n = 1); **Crenuchidae**: *Characidiumfasciatum* Reinhardt, 1867 (n = 24,); *Characidiumzebra* Eigenmann, 1909 (n = 1 paratype); *Characidium* sp. A (n = 91); *Characidium* sp. B (n = 22); *Characidium* sp. C (n = 26); **Bryconidae**: *Bryconopalinus* Cuvier, 1819 (n = 9); **Characidae**: *Phenacogasterfranciscoensis* Eigenmann, 1911 (n = 1), *Astyanaxlacustris* (Lütken, 1875) (n = 221); *Astyanax* sp. (n = 10); *Deuterodon* sp. (8); *Deuterodongiton* (Eigenmann, 1908) (n = 41); *Deuterodonintermedius* (Eigenmann, 1908) (n = 449); *Deuterodonpedri* Eigenmann, 1908(n = 123); Deuterodonaff.taeniatus (n = 395); *Psalidodon* sp. (n = 62); *Psalidodonfasciatus* (Cuvier, 1819) (n = 17); *Psalidodonrivularis* (Lütken, 1875) (n = 765); *Hasemanianana* (Lütken, 1875)(n = 335); *Hasemania* sp. (n = 84); *Knodusmoenkhausii* (Eigenmann & Kennedy, 1903) (n = 662); *Oligosarcusargenteus* Günther, 1864 (n = 153); *Piabinaargentea* Reinhardt, 1867 (n = 8); *Serrapinnusheterodon* (Eigenmann, 1915) (n = 2); **Erythrinidae**: *Hopliasintermedius* (Günther, 1864) (n =) **Aspredinidae**: *Bunocephalushartti* Carvalho, Cardoso, Friel & Reis, 2015 (n = 4); **Trichomycteridae**: *Cambevavariegata* (Costa, 1992) (n = 60); *Trichomycterusalternatus* (Eigenmann, 1917) (n = 522); *Trichomycterusauroguttatus* Costa, 1992 MZUSP 43341 (n = 6); *Trichomycterusbrasiliensis* Lütken, 1874 (n = 27); *Trichomycteruscaudofasciatus* Alencar & Costa, 2004 (n = 21); *Trichomycterusimmaculatus* (Eigenmann & Eigenmann, 1889) (n = 13); *Trichomycterusitacambirussu* Triques & Vono, 2004 (n = 1); *Trichomycterusjequitinhonhae* Triques & Vono, 2004 (n = 3); *Trichomycterusmelanopygius* Reis, dos Santos, Britto, Volpi & de Pinna, 2020 (n = 27); *Trichomycterusnovalimensis* Barbosa & Costa, 2010 (n = 27); *Trichomycteruspauciradiatus* Alencar & Costa, 2006 (n = 7); *Trichomycteruspradensis* Sarmento-Soares, Martins-Pinheiro, Aranda & Chamon, 2005 (n = 43); *Trichomycterusreinhardti* (Eigenmann, 1917) (n = 9); *Trichomycterus* sp. A (n = 16); *Trichomycterus* sp. B (n = 4); **Callichthyidae**: *Callichthyscallichthys* (Linnaeus, 1758) (n = 20); *Hoplosternumlittorale* (Hancock, 1828) Uncatalogued; **Loricariidae**: *Euryochusthysanos* Pereira & Reis, 2017 (n = 10); *Neoplecostomusdoceensis* Roxo, Silva, Zawadzki & Oliveira, 2014 (n = 40); *Neoplecostomusfranciscoensis* Langeani, 1990 (n = 9); *Neoplecostomusparanensis* Langeani, 1990 (n = 73); *Neoplecostomus* sp. A (n = 36); *Neoplecostomus* sp. B (n = 13); *Harttiacarvalhoi* Miranda Ribeiro, 1939 (n = 47); *Harttiagracilis* Oyakawa, 1993 (n = 4); *Harttiaintermontana* Oliveira & Oyakawa, 2019 (n =1); *Harttialeiopleura* Oyakawa, 1993 (n = 2); *Harttialongipinna* Langeani, Oyakawa & Montoya-Burgos, 2001 (n = 1); *Harttialoricariformis* Steindachner, 1877 (n =) ; *Harttianovalimensis* Oyakawa, 1993 (n = 6); *Harttiatorrenticola* Oyakawa, 1993 (n = 81); Harttiacf.gracilis (n = 4); Harttiacf.longipinna (n = 13); *Harttia* sp. (n = 5); *Hypostomusfrancisci* (Lütken, 1874) (n = 4); *Hypostomus* sp. (n = 1); *Pareiorhaphismutuca* (Oliveira & Oyakawa, 1999) (n = 4); *Pareiorhaphisnasuta* Pereira, Vieira & Reis, 2007 (n = 3); *Pareiorhaphisscutula* Pereira, Vieira & Reis, 2010 (n = 119); *Pareiorhaphisvetula* Pereira, Lehmann & Reis, 2016 (n = 25); *Pareiorhaphis* sp. MNRJ 48424 (3); **Heptapteridae**: *Phenacorhamdiatenebrosa* (Schubart, 1964) (n = 1); *Rhamdiaquelen* group (n = 7); **Pimelodidae**: *Duopalatinusemarginatus* (Valenciennes, 1840) (n = 1); *Pimelodusfur* (Lütken, 1864) (n = 1); **Gymnotidae**: *Gymnotuscarapo* group (n = 85); **Sternopygidae**: *Eigenmanniavirescens* (Valenciennes, 1836) (n = 1); **Poeciliidae**: *Phallocerosharpagos* Lucinda, 2008 (n = 269); *Phallocerosuai* Lucinda, 2008 (n = 719); *Poeciliareticulata* Peters, 1859 (n = 234); **Synbranchidae**: *Synbranchusmarmoratus* group (n = 2); **Cichlidae**: *Australoherosmattosi* Ottoni, 2012 (n = 7); *Australoheros* sp. (n = 1); *Geophagusbrasiliensis* (Quoy & Gaimard, 1824) (n = 115).

We recorded 60 species which were distributed in six orders and 17 families (Table 2). Characiformes and Siluriformes were predominant on both sides of the mountain range: 20 and 13 species from the upper rio Santo Antônio, and 17 and 12 species from the upper rio Paraúna. Cyprinodontiformes and Cichliformes were also recorded (three representatives from each of them), followed by Gymnotiformes (two species), and Synbranchiformes (one species). The most representative families were Characidae and Loricariidae, with 12 and seven species, respectively, from the upper rio Santo Antônio; and nine and three species, respectively, from the upper rio Paraúna. The other recorded families were: Anostomidae (eight species); Crenuchidae and Trichomycteridae (four species), Poeciliidae and Cichlidae (three species), Heptapteridae, Pimelodidae and Callichthyidae (two species), Prochilodontidae, Bryconidae, Erythrinidae, Aspredinidae, Gymnotidae, Sternopygidae, and Synbranchidae (one species). Thirty-four species were associated to headwaters of the upper rio Paraúna, while 40 species were attributed to the upper rio Santo Antônio. A total of 14 species was recorded for both drainages. The highest species richness was registered from the ribeirão das Pedras (26 species) and the rio Cipó (17 species) drainages. In the latter was confirmed the highest number of exclusive species (14 species). Three migratory species (*Pimelodusfur*, *Prochiloduscostatus*, and *Megaleporinusobtusidens*); two endangered (*Bryconopalinus* and *Hypomasticusthayeri*); 17 endemic (*Australoherosmattosi*, *Bunocephalushartti*, *Deuterodonpedri*, *Duopalatinusemarginatus*, *Harttiaintermontana*, *Hasemanianana*, *Leporinusmarcgravii*, *L.taeniatus*, *Prochiloduscostatus*, *Pareiorhaphisscutula*, *P.vetula*, *Phallocerosuai*, *Phenacogasterfranciscoensis*, *P.fur*, *Psalidodonrivularis*, *Trichomycterusalternatus*, and *T.melanopygius*); and two exotic species (*Leporinusamblyrhynchus*, and *Poeciliareticulata*) were registered. Four of the aforementioned endemic species (*H.nana*, *P.uai*, *P.rivularis*, and *T.alternatus*) were found out in different basin instead of their original ones. Nineteen species presented some taxonomic inaccuracy and 14 are possibly new species (*Characidium* sp. A, *Characidium* sp. B, *Characidium* sp. C, *Astyanax* sp. A, *Astyanax* sp. B, *Astyanax* sp. C, *Hasemania* sp., *Trichomycterus* sp. A, *Trichomycterus* sp. B, *Harttia* sp., *Neoplecostomus* sp. A, *Neoplecostomus* sp. B, *Pareiorhaphis* sp., and *Australoheros* sp.). Other four species are possibly related to species complex (Deuterodonaff.taeniatus, *Gymnotuscarapo* group, *Rhamdiaquelen* group, and *Synbranchusmarmoratus* group). Historical records of 15 species were obtained exclusively during visits to fish collections (*B.opalinus*, *B.hartii*, *D.emarginatus*, *Eigenmanniavirescens*, *Hoplosternumlittorale*, *Hypostomusfrancisci*, *L.amblyrhynchus*, *L.marcgravii*, *L.taeniatus*, *M.obtusidens*, *P.franciscoensis*, *Phenacorhamdiatenebrosa*, *Piabinaargentea*, *P.fur*, and *P.costatus*).

### Order Characiformes

#### Prochilodus costatus

Taxon classificationAnimaliaCharaciformesProchilodontidae

Valenciennes, 1850

05F29396-D425-5758-B869-6E0F683803AF

##### Distribution.

Rio São Francisco basin.

##### Diagnosis.

*Prochiloduscostatus* is diagnosed from its congeners by having 44–47 perforated scales in the lateral line; 8–9 scales between the origin of dorsal fin and lateral line.

#### Family Anostomidae

##### Hypomasticus mormyrops

Taxon classificationAnimaliaCharaciformesAnostomidae

(Steindachner, 1875)

96E77B53-C641-5753-B216-E5FF45378994

###### Distribution.

Rio Paraíba do Sul, rio Piabanha, and rio Doce basins, Brazil.

###### Diagnosis.

*Hypomasticusmormyrops* differs from *H.thayeri* by the moderate lips; mouth ventral; premaxillary and dentary teeth anteriorly oriented when mouth is closed; first teeth (close to the symphysis in the premaxilla and dentary) larger than the others.

##### Hypomasticus thayeri

Taxon classificationAnimaliaCharaciformesAnostomidae

(Borodin, 1929)

1B76E6BF-D66D-52BC-84DE-4025068746B4

Fig. 3A

###### Distribution.

Rio Paraíba do Sul and rio Jequitinhonha basins, Brazil.

###### Diagnosis.

*Hypomasticusthayeri* differs from *H.mormyrops* by the upper lip developed; mouth subterminal, not facing down; premaxillary teeth posteriorly oriented and dentary teeth anteriorly oriented when mouth is closed; three anterior teeth of premaxilla and dentary with similar size.

**Figure 3.**
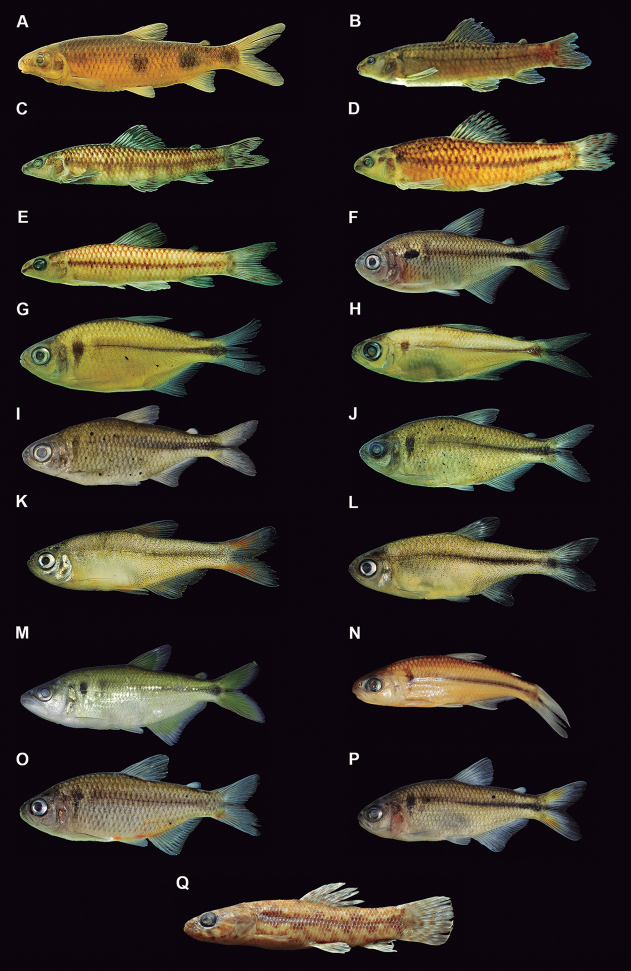
Characiformes species from the middle-southern Espinhaço mountain range, Minas Gerais state, Brazil **A***Hypomasticusthayeri*, MNRJ 43577, 91.1 mm SL **B***Characidiumfasciatum*, MNRJ 48435, 68.8 mm SL **C***Characidium* sp. A, MNRJ 46861, 65.5 mm SL **D***Characidium* sp. B, MNRJ 48460, 56.2 mm SL **E***Characidium* sp. C, MNRJ 46911, 42.3 mm SL **F***Astyanaxlacustris*, MNRJ 48521, 52.6 mm SL **G***Deuterodongiton*, MNRJ 48129, 47.5 mm SL **H***Deuterodonintermedius*, MNRJ 47840, 42.1 mm SL **I***Deuterodonpedri*, MNRJ 48381, 65.5 mm SL **J**Deuterodonaff.taeniatus, MNRJ 45824, 55.5 mm SL **K***Hasemanianana*, MNRJ 48440, 28.4 mm SL **L***Hasemania* sp., MNRJ 48416, 25.1 mm SL **M***Oligosarcusargenteus*, MNRJ 48393, 82.6 mm SL **N***Piabinaargentea*, MZUSP 110200, 44.2 mm SL **O***Psalidodonrivularis*, MNRJ 48516, 46.8 mm SL **P***Psalidodon* sp. MNRJ 48128, 59.4 mm SL **Q***Hopliasintermedius*MZUSP 54696, 40.4 mm SL.

##### Leporellus vittatus

Taxon classificationAnimaliaCharaciformesAnostomidae

(Valenciennes, 1850)

434A5299-894C-5B91-ACB5-8734BA548ED8

###### Distribution.

Rio Amazonas, rio Paraná-Paraguay, and rio São Francisco basins: Brazil, Ecuador, Colombia, Bolivia, Paraguay, and Peru.

###### Diagnosis.

*Leporellusvittatus* is diagnosed by having two longitudinal dark stripes on upper and lower lobes and one on the caudal fin median rays.

##### Leporinus amblyrhynchus

Taxon classificationAnimaliaCharaciformesAnostomidae

Garavello & Britski, 1987

F8E39FF6-8AD4-57F3-A313-C903E90DEA1F

###### Distribution.

Rio Paraná and upper rio São Francisco basins, Brazil.

###### Diagnosis.

*Leporinusamblyrhynchus* differs from its congeners by a longitudinal dark band on the sides of the body and 8–10 transversal dark bands on the dorsal region.

##### Leporinus copelandii

Taxon classificationAnimaliaCharaciformesAnostomidae

Steindachner, 1875

EFAC6900-AA6E-578B-A2C7-4E0DBD73D16D

###### Distribution.

Rio Paraíba do Sul and rio Doce basins, Brazil.

###### Diagnosis.

*Leporinuscopelandii* differs from its congeners by having three rounded or slightly rectangular spots conspicuously distributed in median line of the body, respectively below dorsal fin, below adipose fin and at the end of caudal peduncle; and all fins presenting reddish color.

##### Leporinus marcgravii

Taxon classificationAnimaliaCharaciformesAnostomidae

Lütken, 1875

CF68FA25-04FB-57C3-967B-99328657D193

###### Distribution.

Rio São Francisco basin, Brazil.

###### Diagnosis.

*Leporinusmarcgravii* differs from its congeners by having several conspicuous maculae throughout the lateral line and smaller maculae above and below lateral line; hyaline or slightly darkened fins base.

##### Leporinus taeniatus

Taxon classificationAnimaliaCharaciformesAnostomidae

Lütken, 1875

0639C6A2-06B1-5DBF-A146-AAC50AFBCCBD

###### Distribution.

Rio das Velhas, rio São Francisco basin, Brazil.

###### Diagnosis.

*Leporinustaeniatus* differs from its congeners by dark macula in the maxilla and reddish pigmentation under the longitudinal dark band.

##### Megaleporinus obtusidens

Taxon classificationAnimaliaCharaciformesAnostomidae

(Valenciennes, 1837)

97541093-282A-5852-BA54-E6EE60C3F3A6

###### Distribution.

Upper rio Paraná, rio Jacuí, rio São Francisco, rio Paraguay, and rio Uruguay basins.

#### Family Crenuchidae

##### Characidium fasciatum

Taxon classificationAnimaliaCharaciformesCrenuchidae

Reinhardt, 1867

9DE205DF-C43C-52A0-A523-BC6E3AACF72A

Fig. 3B

###### Distribution.

Rio São Francisco basin.

###### Diagnosis.

*Characidiumfasciatum* differs from *Characidium* sp. A by the high or little tapered snout; adult specimens with vertical bars without defined shape or almost missing in the lateral of the body; narrow longitudinal dark band occupying less than one scale; pigmentation on caudal fin rays not forming conspicuous bands or just forming narrow bands. Distinguished from *Characidium* sp. B by having 36 or 37 perforated scales in the lateral line; four series of scales below lateral line.

##### Characidium

Taxon classificationAnimaliaCharaciformesCrenuchidae

sp. A

F9AA8CE3-894A-5103-8F02-ECBAE14E100A

Fig. 3C

###### Distribution.

Upper rio Santo Antônio, rio Doce basin.

###### Diagnosis.

*Characidium* sp. A differs from its congeners of this study by having very tapered snout; wide and conspicuous vertical bars in the lateral of body in both juveniles and adults specimens; longitudinal dark band occupying one or more scales; one-two dark, wide and conspicuous bands in half of caudal fin rays and another in the base of first and posterior caudal fin ray.

###### Remarks.

In this study, we refer *Characidium* sp. A such as a first putative new species from the rio Doce basin, due to differences in morphology and color pattern between this species and another similar ones from Southeastern Brazil river basins such as *C.alipioi*, *C.grajahuense*, *C.lagosantense*, *C.lauroi*, and *C.timbuiense*.

##### Characidium

Taxon classificationAnimaliaCharaciformesCrenuchidae

sp. B

19061D70-D178-517B-BA01-FB773981726E

Fig. 3D

###### Distribution.

Upper rio Santo Antônio, rio Doce basin.

###### Diagnosis.

*Characidium* sp. B differs from its congeners from the study area by predorsal length less than 45% of total length; lateral vertical bars absent or without defined shape; dark maculae on caudal fin not forming defined bands.

###### Remarks.

In this study, we refer *Characidium* sp. B such as a second putative new species from the rio Doce basin, due to differences in morphology and color pattern between this species and another from Southeastern Brazil river basins such as *C.alipioi*, *C.grajahuense*, *C.lagosantense*, and *C.lauroi*.

##### Characidium

Taxon classificationAnimaliaCharaciformesCrenuchidae

sp. C

9D5AD671-5353-5383-A83C-324C580070B1

Fig. 3E

###### Distribution.

Upper rio Santo Antônio, rio Doce basin.

###### Diagnosis.

*Characidium* sp. C differs from its congeners from the study area by predorsal length up to 55% of total length; vertical bars always arranged above and below the lateral line in a “y” or “yy” shape; weak of narrow dark band on caudal fin.

###### Remarks.

In this study, we refer *Characidium* sp. C such as a third putative new species from the rio Doce basin, due to differences in morphology and color pattern between this species and another from Southeastern Brazil river basins such as *C.cricarense* and *C.litorale*.

#### Family Bryconidae

##### Brycon opalinus

Taxon classificationAnimaliaCharaciformesBryconidae

(Cuvier, 1819)

A2494A3C-DF71-5D83-A5E2-62470178E19E

###### Distribution.

Rio Paraíba do Sul and rio Doce basins.

###### Diagnosis.

*Bryconopalinus* is diagnosed from its congeners by having one humeral spot and another in the caudal peduncle, never extending up to median caudal fin rays; tubules of the lateral line without secondary branches.

#### Family Characidae

##### Astyanax lacustris

Taxon classificationAnimaliaCharaciformesCharacidae

(Lütken, 1875)

B7D241CE-ECC8-582E-ACBA-D083CE02E096

Fig. 3F

###### Distribution.

Rio São Francisco basin, Southeastern Brazil.

###### Diagnosis.

*Astyanaxlacustris* differs from *Astyanax* sp. by the absence of teeth in maxillary bone; a conspicuous oval humeral spot arranged horizontally; hyaline fins usually yellowish, more evident in the caudal fin. It is also diagnosed by having 33–36 perforated scales in lateral line; 26–29 branched rays in anal fin; 6.5–7.5 scales above and 5.5–6.5 scales below lateral line.

##### Astyanax

Taxon classificationAnimaliaCharaciformesCharacidae

sp.

33EBE97C-CFA8-52D7-B8C2-58D1950EF582

###### Distribution.

Upper rio Santo Antônio, rio Doce basin.

###### Diagnosis.

*Astyanax* sp. differs from *A.lacustris* by having teeth in maxillary bone; conspicuous humeral spot vertically oriented; hyaline fins slightly reddish. It is also diagnosed by having teeth tetracuspidate to heptacuspidate in the inner series of premaxillary bone forming a notch; dentary teeth decreasing abruptly in size from fourth tooth; 6.5 scales above the lateral line; iii+19 or 20 anal fin rays.

###### Remarks.

In this study, we refer *Astyanax* sp. such as putative new species from the study area, due to differences in morphology and color pattern between this species and another from Southeastern Brazil river basins such as *A.microschemos* and *A.turmalinensis*.

##### Deuterodon giton

Taxon classificationAnimaliaCharaciformesCharacidae

Eigenmann, 1908

618A64F9-1F3B-5A7C-B929-30A6F222A594

Fig. 3G

###### Distribution.

Rio Paraíba do Sul, Brazil.

###### Diagnosis.

*Deuterodongiton* differs from its congeners of the study area by having dentary teeth decreasing gradually in size until the sixth or seventh tooth; dentary with more than five cusps (usually seven or eight); infraorbital 3 totally exposed, with almost no naked area prior to preopercle; infraorbital 3 shiny due to high concentration of guanine crystals and low concentration of chromatophores.

###### Remarks.

*Deuterodongiton* is described from the rio Paraíba do Sul basin. However, in the present study it was found in the rio Doce basin, confirming the first record of the species for this basin. The difference observed in the specimens between both morphotypes is a tendency of longer length in adult specimens from the rio Paraíba do Sul basin.

##### Deuterodon intermedius

Taxon classificationAnimaliaCharaciformesCharacidae

Eigenmann, 1908

8329C617-3C53-5981-9D1B-32CBA3879CA0

Fig. 3H

###### Distribution.

Rio Paraíba do Sul basin and coastal drainages in state of Rio de Janeiro, Brazil.

###### Diagnosis.

*Deuterodonintermedius* can be distinguished from its congeners of the study area by the absence of space in the symphysis of dentary; five tetracuspidate to hexacuspidate teeth in the inner series of the premaxillae; infraorbital 3 without chromatophores; small humeral spot, sometimes slightly rounded in smaller specimens; no more than 1.5 scales below the lateral line; 35–37 perforated scales in the lateral line

###### Remarks.

*Deuterodonintermedius* is described from the rio Paraíba do Sul basin. However, in the present study it was found in the rio Doce basin. The only difference observed in the specimens between both morphotypes is a tendency of longer length in adult specimens from the rio Paraíba do Sul basin.

##### Deuterodon pedri

Taxon classificationAnimaliaCharaciformesCharacidae

Eigenmann, 1908

B6F79C30-1E67-5745-B34F-2C40E19A0A60

Fig. 3I

###### Distribution.

Rio Doce basin.

##### Deuterodon aff. taeniatus

Taxon classificationAnimaliaCharaciformesCharacidae

BFDABD75-C1CF-582D-8A45-3276518C11D0

Fig. 3J

###### Distribution.

Upper rio Santo Antônio, rio Doce basin.

###### Diagnosis.

Deuterodonaff.taeniatus differs from its congeners of the study area by the presence of a space in the symphysis of dentary; infraorbital 3 with high concentration of chromatophores; usually verticalized humeral spot with a lower comma-shaped feature, reaching 2.5 scales below the lateral line; 32–39 perforated scales in the lateral line; iii-v+17–24 anal fin rays.

###### Remarks.

*Deuterodontaeniatus* is described from the rio São João and rio Macaé basins (rio Paraíba do Sul basin), in state of Rio de Janeiro, Brazil. The presence of the species in different basins has been notified in literature (Vieira 2006; Alves and Pompeu 2010) and sometimes with imprecise taxonomy (e.g., Alves et al. 2008; Vieira et al. 2015). In this study, *A.taeniatus* was recorded only for the rio Doce basin. However, it was observed that specimens from the rio Doce basin have lower body depth when compared to the morphotypes from rio Paraíba do Sul basin.

##### Deuterodon

Taxon classificationAnimaliaCharaciformesCharacidae

sp.

3E3715FD-7F36-5029-B838-6882CA724CC2

###### Distribution.

Upper rio Santo Antônio, rio Doce basin.

###### Diagnosis.

*Deuterodon* sp. differs from its congeners from the study area by having five hexacuspidate to heptacuspidate teeth in the inner series of the premaxillae; cusps straight, not forming notch; infraorbital 3 with naked area anteriorly, and below it; low concentration of chromatophores in the infraorbital 3; inconspicuous humeral spot slightly verticalized, straight anteriorly and straight or half-moon shaped posteriorly; 5.5 scales above lateral line; iii+21 anal fin rays.

###### Remarks.

In this study, we refer *Deuterodon* sp. such as putative new species from the rio Doce basin, due to differences in morphology and color pattern between this species and another from southeastern Brazil river basins such as *D.giton*, *D.intermedius*, and *D.taeniatus*.

##### Hasemania nana

Taxon classificationAnimaliaCharaciformesCharacidae

(Lütken, 1875)

9C69093A-15DC-5F9F-9E10-D001036B926E

Fig. 3K

###### Distribution.

Rio São Francisco basin.

###### Diagnosis.

*Hasemanianana* differs from *Hasemania* sp. by having 13–19 branched rays in anal fin and absence of rounded blotch in the median caudal fin rays.

###### Remarks.

*Hasemanianana* is an endemic species from the rio São Francisco basin. However, in the present study it was found in the rio Doce basin, confirming the first record of the species for this basin. The only difference observed in the specimens between both morphotypes is a tendency of higher number of anal fin rays in the specimens from the rio Doce basin (13–19 vs. 13–16). The higher number of anal fin rays is congruent to the *H.nana* morphotype from the rio Paraopeba (rio São Francisco basin).

##### Hasemania

Taxon classificationAnimaliaCharaciformesCharacidae

sp.

7C343141-CF0F-5FA3-8226-B91D5AD493B9

Fig. 3L

###### Distribution.

Upper rio Paraúna, rio São Francisco basin.

###### Diagnosis.

*Hasemania* sp. differs from *H.nana* by having 11–14 branched rays in anal fin and presence of rounded blotch in the base of median caudal fin rays.

###### Remarks.

In this study, we refer *Hasemania* sp. such as putative new species and second record from the rio São Francisco basin, due to differences in morphology and color pattern between this species and another from Southeastern Brazil river basins such as *H.bilineata*, *H.crenuchoides*, *H.nana*, and *H.uberaba*. In the taxonomic revision carried out by Serra (2003) there is no mention of the morphotype. The same have occurred in Vieira et al. (2015) resulting here in the first record of the taxon for the basin.

##### Knodus moenkhausii

Taxon classificationAnimaliaCharaciformesCharacidae

(Eigenmann & Kennedy, 1903)

2745CC2C-4939-5500-A768-FEF1EB3591D6

###### Distribution.

Rio Doce, rio Paraíba do Sul, upper rio Paraná, rio Paraguay, and rio Jequitinhonha basins, in Brazil, and some drainages in Bolivia and Paraguay.

###### Remarks.

Occurrence of *K.moenkhausii* in the rio Doce basin was already confirmed. Different studies have mentioned about such record (dos Santos 2015; Vieira et al. 2015; Sales et al. 2018). In Vieira et al. (2015) the species was identified such as K.cf.moenkhausii. In tributaries of the rio Santo Antônio basin is quite common to collect it. However, it may be an exotic species which was introduced on the basin in the past (Vieira et al. 2015), but future research will be needed to confirm how the species reached the basin. Souza et al. (2015) revealed through DNA barcoding technique that *K.moenkhausii* has been shared throughout rio São Francisco, rio Paraíba do Sul and upper rio Paraná basins, through recent interchange. According to the authors, the species represent a single panmitic species, and its sharing in those basins may have occurred due to different human activity processes, such as intentional introduction, transposition of natural barriers or accidental escape in ornamental fish trade.

##### Oligosarcus argenteus

Taxon classificationAnimaliaCharaciformesCharacidae

Günther, 1864

E013586C-0561-5B69-9F23-0873178C5CA7

Fig. 3M

###### Distribution.

Rio Doce, rio das Velhas, and upper rio Paraná basins, Brazil.

###### Diagnosis.

*Oligosarcusargenteus* is diagnosed by having 17–24 teeth in maxillary bone; 44–48 perforated scales in lateral line; 8–9 series of scales above and 6–8 below lateral line; iv-v+20–25 branched rays in the anal fin; 17–20 scales around caudal peduncle.

##### Phenacogaster franciscoensis

Taxon classificationAnimaliaCharaciformesCharacidae

Eigenmann, 1911

44B218E3-1D9A-5AD0-9ED7-E1E330EAA397

###### Distribution.

Rio São Francisco basin, Brazil.

##### Piabina argentea

Taxon classificationAnimaliaCharaciformesCharacidae

Reinhardt, 1867

CDC74734-CEEE-5450-8B5D-3CBD50B561E3

Fig. 3N

###### Distribution.

Upper rio Paraná, rio São Francisco, rio Itapicuru, rio Paraíba do Sul, and rio Itapemirim basins: Brazil and Paraguay.

###### Diagnosis.

*Piabinaargentea* is diagnosed by having longitudinal dark band in the lateral of the body; dark spot in caudal peduncle absent; 18–21 rays in anal fin.

##### Psalidodon rivularis

Taxon classificationAnimaliaCharaciformesCharacidae

(Lütken, 1875)

EFBB8D80-BCB6-5D4E-9092-7B6C1C442F52

Fig. 3O

###### Distribution.

Rio São Francisco basin, Southeastern Brazil.

###### Diagnosis.

*Psalidodonrivularis* differs from its congeners of the study area by having premaxilla aligned with dentary in lateral view; four or five wide teeth in the inner series of premaxilla (if present, the fifth tooth is too small or not aligned with others); chromatophores surrounding abdominal scales and in higher concentration on the base of scales; developed scales in pectoral, pelvic, and anal fins.

###### Remarks.

*Psalidodonrivularis* was originally described as an endemic species from the rio São Francisco basin. However, in the present study it was found in the rio Doce basin, confirming record of the species in this basin. Oliveira (2017) also confirmed the occurrence of *P.rivularis* as such as from the headwaters of rio Doce plus the headwaters of rio Jequitinhonha basin, while suggesting the synonymy of *Astyanaxturmalinensis* (Triques, 2003) with *Psalidodonrivularis*.

##### Psalidodon

Taxon classificationAnimaliaCharaciformesCharacidae

sp.

5205CA9E-D13C-5856-B1E0-A8D2076A503D

Fig. 3P

###### Distribution.

Upper rio Paraúna, rio São Francisco basin, and upper rio Santo Antônio, rio Doce basin.

###### Diagnosis.

*Psalidodon* sp. can be distinguished from its congeners from the study area, except of *P.rivularis*, by higher body anteriorly to dorsal fin origin; from *A.rivularis* by having premaxilla slightly in front of dentary in lateral view; five narrow teeth aligned in the inner series of premaxillary bone; two narrow vertical lines of chromatophores surrounding border of abdominal scales; small hooks in pectoral and anal fins in mature males.

###### Remarks.

In this study, we refer *Psalidodon* sp. such as putative new species from the study area, due to differences in morphology and color pattern between this species and another Stethaprioninae from Southeastern Brazil river basins, such as *P.fasciatus*, *P.rivularis*, *A.scabripinnis*, and *A.turmalinensis*.

##### Hoplias intermedius

Taxon classificationAnimaliaCharaciformesCharacidae

(Günther, 1864)

A79FB3C0-0D4C-50A3-8E48-CE1812008C61

Fig. 3Q

###### Distribution.

Rio São Francisco and rio Paraná basins plus tributaries of the rio Doce, Brazil.

###### Diagnosis.

*Hopliasintermedius* is diagnosed by having 4–6 pores in lateral sensory system of the ventral surface of dentary; 42–46 perforated scales in lateral line; dark or light brown color in head and body.

### Order Siluriformes

#### Family Aspredinidae

##### Bunocephalus hartii

Taxon classificationAnimaliaSiluriformesAspredinidae

Carvalho, Cardoso, Friel & Reis, 2015

523AFB0F-8798-57DE-A647-B13C3BAB685A

Fig. 4A

###### Distribution.

Middle rio São Francisco basin, Minas Gerais, Brazil.

###### Diagnosis.

*Bunocephalushartii* is diagnosed by the absence of hooks throughout anterior margin of spine of pectoral fin; posterior ray of dorsal fin completely or almost adnate to dorsum.

**Figure 4.**
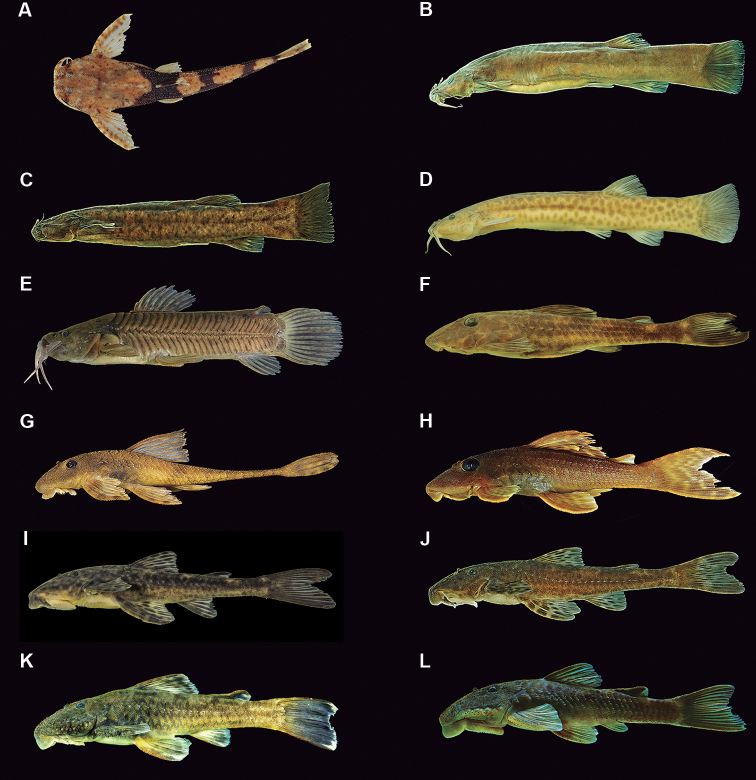
Siluriformes species from the middle-southern Espinhaço mountain range, Minas Gerais state, Brazil **A***Bunocephalushartti*, MZUSP 064227, 44.7 **B***Trichomycterusmelanopygius*, MNRJ 47902, 85.2 mm SL **C***Trichomycterus* sp. A, MNRJ 47901, 87.4 mm SL **D***Trichomycterus* sp. B, MNRJ 46932, 57.0 mm SL **E***Callichthyscallichthys*, MNRJ 48501, 58.2 mm SL **F***Euryochusthysanos*, MNRJ 47897, 74.4 mm SL **G***Harttiaintermontana*, MNRJ 48463, 42.4 mm SL **H***Hypostomusfrancisci*, MZUSP 37162, 66.8 mm SL **I***Neoplecostomus* sp. A, MNRJ 46935, 73.0 mm SL **J***Neoplecostomus* sp. B, MNRJ 48431, 43.0 mm SL **K***Pareiorhaphisscutula*, MNRJ 48471, 88.1 mm SL **L***Pareiorhaphisvetula*, MNRJ 46936, 40.4 mm SL.

#### Family Trichomycteridae

##### Trichomycterus alternatus

Taxon classificationAnimaliaSiluriformesTrichomycteridae

(Eigenmann, 1911)

FD49DC56-24EF-5FA8-8322-D1AE67CBBFDD

###### Distribution.

Rio Doce basin, in the states of Minas Gerais and Espírito Santo, Brazil.

###### Diagnosis.

*Trichomycterusalternatus* differs from its congeners of the rio Doce and rio São Francisco basins by having seven branchiostegal rays; yellowish to light brown body color; rectangular or rounded sequential dark maculae at the midline of the body, sometimes fused and with a vermicular pattern, or forming a narrow stripe from the post-opercular region to the base of caudal fin; a row of rectangular sequential middorsal maculae, round or fused to maculae of the midlateral of the body; subtruncate caudal fin.

##### Trichomycterus melanopygius

Taxon classificationAnimaliaSiluriformesTrichomycteridae

Reis, dos Santos, Britto, Volpi & de Pinna, 2020

2BE880ED-E3AD-5803-8E5B-C280E55F9DD8

Fig. 4B

###### Distribution.

Tributaries from rio Doce basin.

###### Diagnosis.

*Trichomycterusmelanopygius* differs from its congeners of the study area by the absence of evident maculae, spots, streaks and/or stripes on the flanks and dorsum of the body; i+7 (rarely i+8) pectoral fin rays; dark band in the median caudal fin rays.

##### Trichomycterus

Taxon classificationAnimaliaSiluriformesTrichomycteridae

sp. A

22F173E9-6F28-5A6E-9A0B-90B5E1C4A882

Fig. 4C

###### Distribution.

Upper rio Santo Antônio, rio Doce basin.

###### Diagnosis.

*Trichomycterus* sp. A is distinguished from its congeners of the study area by the spotted body due to high concentration of large maculae with no defined shape; caudal fin strongly truncated; eight branchiostegal rays; few dorsal procurrent rays (14 or 15).

###### Remarks.

In this study, we refer *Trichomycterus* sp. A such as a putative new species from the rio Doce basin, due to differences in morphology and color pattern between this species and another with similar color pattern from Southeastern Brazil river basins such as *T.auroguttatus*, *T.albinotatus*, *T.brasiliensis* group, *T.caipora*, *T.argos*, *T.novalimensis*, and *T.rubiginosus*.

##### Trichomycterus

Taxon classificationAnimaliaSiluriformesTrichomycteridae

sp. B

D69E368E-43A0-54C0-B6D9-DACD603F1610

Fig. 4D

###### Distribution.

Upper rio Paraúna, rio São Francisco basin, and upper rio Santo Antônio, rio Doce basin.

###### Diagnosis.

*Trichomycterus* sp. B differs from its congeners of the study area by having nine branchiostegal rays; high concentration of rounded dark spots on the head and sides of the trunk, dorsum, and belly, which may fuse and form small vermiculations; rounded caudal fin.

###### Remarks.

In this study, we refer *Trichomycterus* sp. B such as a putative new species from the rio Doce basin, due to differences in morphology and color pattern between this species and another with similar color pattern from Southeastern Brazil river basins such as *T.brasiliensis* group, *T.argos*, *T.landinga*, *T.novalimensis*, and *T.rubiginosus*.

#### Family Callichthyidae

##### Callichthys callichthys

Taxon classificationAnimaliaSiluriformesCallichthyidae

(Linnaeus, 1758)

BC789CA9-3135-5E6F-8EC5-A6F40F9AE404

Fig. 4E

###### Distribution.

Drainages from Colombia to the Río de La Plata basin, South America.

##### Hoplosternum littorale

Taxon classificationAnimaliaSiluriformesCallichthyidae

(Hancock, 1828)

F271F57C-3784-5483-BBC6-C42785DE7B52

###### Distribution.

Widespread in South America.

#### Family Loricariidae

##### Euryochus thysanos

Taxon classificationAnimaliaSiluriformesLoricariidae

Pereira & Reis, 2017

B0BCB7DD-3022-5CE3-BB2B-1AAB2CFECB04

Fig. 4F

###### Distribution.

Coastal rivers in Eastern Brazil, from the rio Itapemirim, and including the larger basins of the rio Doce and Mucuri, in Espirito Santo and Minas Gerais states, to the rio Frades, state of Bahia.

###### Diagnosis.

*Euryochusthysanos* is diagnosed by having rounded and short inferior lip, leaving a large naked area in the ventral portion of head; inferior lip with barbel developed; 30–35 bicuspidate teeth in the premaxillary and dentary bones; absence of hypertrophied odontodes.

##### Harttia

Taxon classificationAnimaliaSiluriformesLoricariidae

sp.

C9969084-37AE-58E9-A739-D38EAFB7E7D8

###### Distribution.

Upper rio Santo Antônio, rio Doce basin.

###### Diagnosis.

*Harttia* sp. differs from *H.intermontana* by inferior region of orbit straight; compressed and narrow plates with developed odontodes in the dorsal and ventral region of caudal peduncle.

###### Remarks.

In this study, we refer *Harttia* sp. such as a putative new species from the rio Doce basin, due to differences in morphology between this species and another from Southeastern Brazil river basins such as *H.carvalhoi*, *H.garavelloi*, *H.leiopleura*, *H.loricariformis*, *H.novalimensis*, and *H.torrenticola*.

##### Harttia intermontana

Taxon classificationAnimaliaSiluriformesLoricariidae

Oliveira & Oyakawa, 2019

E62C1C5D-C8C3-511B-93A1-4EA4325B5A1D

Fig. 4G

###### Distribution.

Upper rio Doce basin, Brazil.

###### Diagnosis.

*Harttiaintermontana* differs from *Harttia* sp. by having orbit rounded; short and wide plates with poorly developed odontodes in the dorsal and ventral region of caudal peduncle.

##### Hypostomus

Taxon classificationAnimaliaSiluriformesLoricariidae

sp.

3DAEE0BF-18E1-5C4A-BF54-40C11A966D16

###### Distribution.

Upper rio Santo Antônio, rio Doce basin.

###### Diagnosis.

*Hypostomus* sp. differs from *H.francisci* by having black and large spots in the head and throughout the body.

###### Remarks.

The only juvenile specimen collected in the study area was analyzed in such a way that is not possible to mention about species level identity or if it configures into a new species.

##### Hypostomus francisci

Taxon classificationAnimaliaSiluriformesLoricariidae

(Lütken, 1874)

42DF86E5-AED6-5C99-A79C-C4462E1699E9

Fig. 4H

###### Distribution.

Rio São Francisco and rio Paraná basins.

###### Diagnosis.

*Hypostomusfrancisci* can be distinguished from *Hypostomus* sp. by pale small, rounded spots in the whole body, including in the fins; spine of the dorsal fin slightly smaller than predorsal distance.

##### Neoplecostomus

Taxon classificationAnimaliaSiluriformesLoricariidae

sp. A

8EB4F228-B815-5D4B-BFDD-22E43949A93E

Fig. 4I

###### Distribution.

Upper rio Santo Antônio, rio Doce basin.

###### Diagnosis.

*Neoplecostomus* sp. A differs from *Neoplecostomus* sp. B by the maxillary barbels poorly developed; premaxillary teeth and dentary with separate cusps and large concavity between them; lateral and central cusps with similar size; no developed papillae between branches of dentary; plates between dorsal and adipose fin meeting on the back of the dorsum.

###### Remarks.

In this study, we refer *Neoplecostomus* sp. A such as a putative new species from the rio Doce basin, due to differences in morphology between this species and another from the rio Doce basin such as *N.doceensis*, and *N.pirangaensis*.

##### Neoplecostomus

Taxon classificationAnimaliaSiluriformesLoricariidae

sp. B

FE672EB4-0205-5A49-9EB3-8B5496961750

Fig. 4J

###### Distribution.

Upper rio Paraúna, rio São Francisco basin.

###### Diagnosis.

*Neoplecostomus* sp. B differs from *Neoplecostomus* sp. A by the maxillary barbels developed; premaxillary teeth and dentary with close cusps; median cusp more developed than lateral one; plates between dorsal and adipose fin not meeting.

###### Remarks.

In this study, we refer *Neoplecostomus* sp. B such as a putative new species from the rio São Francisco basin, due to differences in morphology between this species and another from rio São Francisco basin such as *N.franciscoensis*.

##### Pareiorhaphis scutula

Taxon classificationAnimaliaSiluriformesLoricariidae

Pereira, Vieira & Reis, 2010

238B295F-CEA5-5E70-95CB-77E04A3C64EE

Fig. 4K

###### Distribution.

Upper rio Doce basin, Brazil.

###### Diagnosis.

*Pareiorhaphisscutula* differs from its congeners of the study area by having abdomen with small plates covered by skin from the pectoral fin region to insertion of pelvic fins; fins with pale yellow and light brown spots.

##### Pareiorhaphis vetula

Taxon classificationAnimaliaSiluriformesLoricariidae

Pereira, Lehmann & Reis, 2016

039EDB19-AF08-5B8F-AE3A-92E62564CA6E

Fig. 4L

###### Distribution.

Rio Doce basin, Brazil.

###### Diagnosis.

*Pareiorhaphisvetula* can be distinguished from its congeners of the rio Doce basin by inferior lip with maxillary barbels completely adnate; elongated and pointed urogenital papilla.

##### Pareiorhaphis

Taxon classificationAnimaliaSiluriformesLoricariidae

sp.

2EDDE782-C26F-58F0-8152-6068207DC94E

###### Distribution.

Upper rio Paraúna, rio São Francisco basin.

###### Diagnosis.

*Pareiorhaphis* sp. differs from *Pareiorhaphisscutula* by the abdomen without plates; from *P.scutula* and *P.vetula* by the pectoral, pelvic and anal fins with clear color; dark caudal with clear borders. *Pareiorhaphis* sp. can still be distinguished from *Pareiorhaphisvetula* by inferior lip with developed maxillary barbels; urogenital papilla with normal size, not elongated.

###### Remarks.

In this study, we refer *Pareiorhaphis* sp. such as a putative new species from the rio Doce basin, due to differences in morphology between this species and another from rio Doce basin such as *P.nasuta*, and *P.proskynita*.

#### Family Heptapteridae

##### Phenacorhamdia tenebrosa

Taxon classificationAnimaliaSiluriformesHeptapteridae

(Schubart, 1964)

2A118B81-B695-53E9-9D85-37F4DB711F4E

###### Distribution.

Upper rio Paraná and rio São Francisco basins, Argentina and Brazil.

##### Rhamdia quelen

Taxon classificationAnimaliaSiluriformesHeptapteridae

group

AF9DE010-E497-5DEF-A0A4-594ECD16DC66

###### Distribution.

Coastal river drainages from state of Rio de Janeiro to state of Santa Catarina, Brazil (Angrizani & Malabarba, 2020).

###### Remarks.

A redescription of *R.quelen* was made, and the original locality where it comes from is rio Macacu drainage, a tributary of rio Soarinho, in the municipality of Cachoeira de Macacu, state of Rio de Janeiro, Brazil.

#### Family Pimelodidae

##### Pimelodus fur

Taxon classificationAnimaliaSiluriformesPimelodidae

(Lütken, 1874)

23F08E4B-249E-594C-B49B-FBA0BFBFCA37

###### Distribution.

Rio das Velhas drainages in rio São Francisco basin, Brazil.

##### Duopalatinus emarginatus

Taxon classificationAnimaliaSiluriformesPimelodidae

(Valenciennes, 1840)

A00A80BD-BC5D-5DFC-9F45-EE258067AF7B

###### Distribution.

Rio São Francisco basin, Brazil.

### Order Gymnotiformes

#### Family Gymnotidae

##### Gymnotus carapo

Taxon classificationAnimaliaGymnotiformesGymnotidae

group

AEA8BF57-4772-51A7-A123-A514D0ACCD14

Fig. 5A

###### Distribution.

Upper rio Paraúna, rio São Francisco basin, and upper rio Santo Antônio, rio Doce basin.

###### Diagnosis.

*Gymnotuscarapo* group is diagnosed by having the mouth upturned, pronate; rictus curved ventrally; eyes positioned below half of median line of the head; branchial opening throughout the posterior margin of opercle; oblique and conspicuous dark bands in the lateral of the body, from dorsal region to ventral surface of preanal; longitudinal band reaching the base of the anal fin.

###### Remarks.

Although *G.carapo* is widespread from Trinidad and Tobago to Argentina, in this study we refer the species as *G.carapo* group, since the taxonomic status of *G.carapo* is uncertain for the Southeastern and Southern Brazil and may represent more than one species. A taxonomic review of the *G.carapo* group in Central and South America is needed.

**Figure 5.**
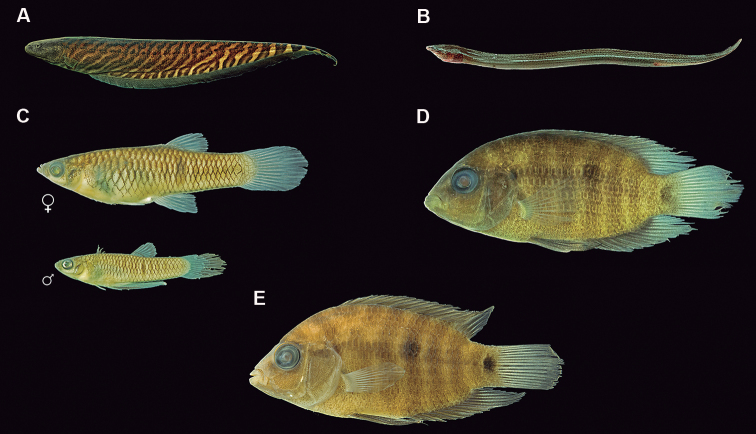
Fish species from the middle-southern Espinhaço mountain range, Minas Gerais state, Brazil **A**Gymnotiformes (*Gymnotuscarapo* group, MNRJ 48407, 130.5 mm SL) **B**Synbranchiformes (*Synbranchusmarmoratus* group, MNRJ 48448, 116.8 mm SL) **C**Cyprinodontiformes (*Phallocerosuai*, MNRJ 48408, 32.0 mm (female – above) and 16.3 mm (male – below) SL) **D**Cichliformes (*Australoherosmattosi*, MNRJ 48454, 54.6 mm SL **E**Cichliformes (*Australoheros* sp., MNRJ 46859, 39.3 mm SL).

#### Family Sternopygidae

##### Eigenmannia virescens

Taxon classificationAnimaliaGymnotiformesSternopygidae

(Valenciennes, 1836)

C26726AF-818C-54C6-9B7D-4707A0DE3711

###### Distribution.

Widespread from rio Orinoco to rio de La Plata basin.

###### Diagnosis.

*Eigenmanniavirescens* is diagnosed by having small branchial opening, smaller than the snout; body light brown, maculae absent.

### Order Synbranchiformes

#### Family Synbranchidae

##### Synbranchus marmoratus

Taxon classificationAnimaliaSynbranchiformesSynbranchidae

group

E5D67FF4-D259-5FF0-8F57-52DEAA8B6FD4

Fig. 5B

###### Distribution.

Upper rio Santo Antônio, rio Doce basin.

###### Diagnosis.

*Synbranchusmarmoratus* group is diagnosed by having dark-brown color on dorsal region of body and light brown below the midline and head; many rounded dark spots or irregularly shaped dark spots throughout the whole body; dark stripe composed by fusion of many dark spots in ventrolateral region of head.

###### Remarks.

Albeit the true *S.marmoratus* have a wide distribution in the Neotropical region, in this study we refer the species as *S.marmoratus* group, since the taxonomic status of *S.marmoratus* may represent more than one species. A taxonomic review of *S.marmoratus* from Central and South America is needed to clarify this problematic issue.

### Order Cyprinodontiformes

#### Family Poeciliidae

##### Phalloceros harpagos

Taxon classificationAnimaliaCyprinodontiformesPoeciliidae

Lucinda, 2008

ABDF2D3D-D745-529A-B1F4-5EA357076475

###### Distribution.

Rio Paraná-Paraguay basin and coastal rivers from rio Itabapoana to rio Araranguá, in Brazil, Paraguay, and Argentina.

###### Diagnosis.

*Phallocerosharpagos* differs from *P.uai* by having gonopodium with hook in the two gonopodial appendages in males positioned close to the appendages; distal half of the appendage narrower than proximal half; urogenital papilla centralized in females, positioned between anus and the base of first anal fin ray.

###### Remarks.

Albeit *P.harpagos* presents a relatively wide distribution in coastal drainages of Brazil, here we present the first record of the species in the rio Santo Antônio basin.

##### Phalloceros uai

Taxon classificationAnimaliaCyprinodontiformesPoeciliidae

Lucinda, 2008

D5751C61-FA0A-5473-962C-2A00217E5E05

Fig. 5C

###### Distribution.

Rio das Velhas, rio São Francisco basin, Brazil.

###### Diagnosis.

*Phallocerosuai* differs from *P.harpagos* by having gonopodium with small left hook facing down or up and forward in males, situated near to gonopodial appendage base; right hook absent; urogenital papilla directed to the right side in females, positioned laterally; anal opening in contact with first anal fin ray or close to it.

###### Remarks.

*Phallocerosuai* is an endemic species from rio São Francisco basin and, in this study, we present the first record for the rio Santo Antônio basin. Vieira et al. (2015) recorded the species in the rio Piracicaba, another important drainage from the rio Doce basin. The distribution of the species in adjacent basin deserves further research, and it is still being carefully investigated.

##### Poecilia reticulata

Taxon classificationAnimaliaCyprinodontiformesPoeciliidae

Peters, 1859

6B1FC357-DDD7-5BB3-A1AF-FE5BC846FB68

###### Distribution.

Trinidad and Tobago in Central America and Northern South America.

###### Remarks.

*Poeciliareticulata* was widely introduced in aquatic environments in Brazil, so its occurrence in many rivers of the country is quite common nowadays.

### Order Cichliformes

#### Family Cichlidae

##### Australoheros mattosi

Taxon classificationAnimaliaCichliformesCichlidae

Ottoni, 2012

769FCF87-9237-58E2-96F5-E4A063004054

Fig. 5D

###### Distribution.

Rio São Francisco basin, Southeastern Brazil.

###### Diagnosis.

*Australoherosmattosi* differs from *Australoheros* sp. by having XVI–XVII+9–11 dorsal fin rays; VII–VIII+9 anal fin rays.

##### Australoheros

Taxon classificationAnimaliaCichliformesCichlidae

sp.

E7048741-60F3-5AE0-B379-AA9E200904EC

Fig. 5E

###### Distribution.

Upper rio Santo Antônio, rio Doce basin.

###### Diagnosis.

*Australoheros* sp. differs from *A.mattosi* by having XVIII+7 dorsal fin rays; VIII+7 anal fin rays.

###### Remarks.

In this study, we refer *Australoheros* sp. such as putative new species due to differences in morphology between it and another from rio Doce basin, since *A.perdi* and *A.ipatinguensis* seems to be restricted to small areas such as lagoons and small rivers in the rio Doce basin. However, a higher number of specimens with difference sizes is needed and a taxonomic review of the cichlid *Australoheros* genus as well.

##### Geophagus brasiliensis

Taxon classificationAnimaliaCichliformesCichlidae

(Quoy & Gaimard, 1824)

FDC8DB3F-515E-58CE-B924-84CA76206611

###### Distribution.

coastal drainages in eastern Brazil.

An identification key for its species from the study area is provided.

### Identification key to orders

**Table:** 

1	body covered by scales	**2**
–	scales absent, naked body covered by skin or bony plates	**5**
2	pelvic, dorsal, and caudal fins absent	** GYMNOTIFORMES **
–	pelvic, dorsal, and caudal fins present	**3**
3	presence of spines in pelvic, dorsal, and anal fins	** CICHLIFORMES **
–	spines in pelvic, dorsal, and anal fins absent	**4**
4	premaxilla protractible; anal fin modified in gonopodium in males; adipose fin absent	** CYPRINODONTIFORMES **
–	premaxilla non-protractible; anal fin not modified in gonopodium in males; adipose fin usually present	** CHARACIFORMES **
5	barbels present on anterior portion of head (at least, one pair); two branchial openings located in variable position in the head; pelvic and pectoral fins present	** SILURIFORMES **
–	barbels absent; single branchial opening, located ventrally in the head; pelvic and pectoral fins absent	**SYNBRANCHIFORMES (Synbranchidae: 1 gen., 1 sp.) (*Synbranchusmarmoratus* group)**

### Identification keys to families

CHARACIFORMES

**Table:** 

1	Small teeth implanted in thick moving lips in premaxillary and dentary bones	**Prochilodontidae (*Prochiloduscostatus*)**
–	Teeth with different shapes, sizes, and numbers in premaxilla and dentary, implanted in bones of the maxillae	**2**
2	Fontanel absent in head	**3**
–	Fontanel present	**4**
3	A single series of conical teeth in premaxillary and dentary bones; presence of three unbranched rays in the pectoral fins; caudal fin forked	** Crenuchidae **
–	Canine teeth; one unbranched ray in the pectoral fins; rounded caudal fin (with rounded margin)	**Erythrinidae (*Hopliasintermedius*)**
4	Supraorbital bone present; 6–8 recurved teeth in a single series in premaxillary and dentary bones, decreasing in size from symphysis to corner of mouth, and arranged as a ladder; teeth close to the symphysis conspicuously larger than the lateral ones; gill membranes attached to the isthmus	** Anostomidae **
–	Supraorbital bone absent; 1–3 series of cuspidate teeth in premaxilla and 1–2 series in dentary, cusps present or not; branchial membranes free from isthmus	** Characidae **

SILURIFORMES

**Table:** 

1	body totally or partially covered by bone plates	**2**
–	body covered by skin; bony plates absent	**3**
2	Mouth terminal or subterminal; double series of plates in the sides of body; nuchal plate meeting parieto-supraoccipital bone in the midline of predorsal region	** Callichthyidae **
–	Mouth ventral, forming an oral disc; presence of plates on each side of body arranged in three longitudinal series or more	** Loricariidae **
3	presence of patch of odontodes in preopercle and opercle	** Trichomycteridae **
–	odontodes absent	**4**
4	head and body severely depressed, its maximum width at posterior region of skull and pectoral girdle; adipose fin absent	**Aspredinidae (*Bunocephalushartti*)**
–	slightly depressed or rounded head and high body; adipose fin present	**5**
5	head higher than wide; first ray of pectoral and dorsal fins modified in an acute and penetrating spine	** Pimelodidae **
–	head as wide as high; first ray of pectoral and dorsal fins modified in a hard spine, but not exactly an acute and penetrating spine	** Heptapteridae **

GYMNOTIFORMES

**Table:** 

1	Mouth terminal; narrow head; frontal and parietal fontanels present; anal fin not reaching the posterior end of the body	**Sternopygidae (*Eigenmanniavirescens*)**
–	Mouth upturned; wide head; frontal fontanel absent; anal fin reaching the posterior margin of body	**Gymnotidae (*Gymnotuscarapo* group)**

CYPRINODONTIFORMES

**Table:** 

1	Poecillidae (1 subfamily)
–	Third, fourth and fifth rays of the anal fin modified in an intromittent organ (gonopodial structure)	** Poecilliinae **


CICHLIFORMES

**Table:** 

1	Presence of spines and soft rays in dorsal, pelvic and anal fins; lateral line divided in anterior and posterior branches: one located in the laterodorsal region of flank (from posterior region of opercle to caudal peduncle region); and the other located in a median line (from caudal peduncle to the base of the caudal fin	** Cichlidae **

### Identification keys to genera and species

FAMILY ANOSTOMIDAE

**Table:** 

1	dark blotch on anterior dorsal fin rays	***Leporellus* (*Leporellusvittatus*)**
–	Absence of dark blotch on dorsal fin rays and dark stripes on caudal fin lobes	**2**
2	dark longitudinal band present; absence of three or more large maculae in the sides of the body	**3**
–	dark longitudinal band absent; three or more large maculae in the lateral of body	**4**
3	dark macula in the maxilla; reddish pigmentation under the longitudinal band	*** Leporinus taeniatus ***
–	dark macula in the maxilla absent; 8–10 transversal dark bands in the dorsal region	*** Leporinus amblyrhynchus ***
4	terminal mouth	**5**
–	Mouth subterminal or ventral; premaxilla ventrally oriented	**7**
5	premaxilla and dentary with three teeth	*** Megaleporinus obtusidens ***
–	premaxilla and dentary with 4 teeth; red macula in the mouth commissure	**6**
6	three rounded or slightly rectangular spots conspicuously distributed in median line of the body, respectively below dorsal fin, below adipose fin and at the end of caudal peduncle; all fins presenting reddish color	*** Leporinus copelandii ***
–	several conspicuous maculae throughout the lateral line and smaller maculae above and below lateral line; hyaline fins or slightly darkened on base	*** Leporinus marcgravii ***
7	anterior region of snout convex in lateral view, moderate lips; mouth ventral; premaxillary and dentary teeth anteriorly oriented when mouth closed; first teeth (close to the symphysis in the premaxilla and dentary) larger than the others	*** Hypomasticus mormyrops ***
–	anterior region of snout straight in lateral view, upper lip developed; mouth subterminal, not facing down; premaxillary teeth posteriorly oriented and dentary teeth anteriorly oriented when mouth closed; three anterior teeth of premaxilla and dentary with similar size	*** Hypomasticus thayeri ***


FAMILY CRENUCHIDAE

**Table:** 

1	very tapered snout; wide and conspicuous vertical bars in the lateral of the body in both juveniles and adult specimens; longitudinal dark band occupying one or more scales; one to two dark, wide and conspicuous bands in half of caudal fin rays and another in the base of first and posterior caudal fin ray	***Characidium* sp. A**
–	high or little tapered snout; adult specimens with vertical bars without defined shape or almost missing in the lateral of the body; narrow longitudinal dark band occupying less than one scale; pigmentation on caudal fin rays not forming conspicuous bands or just forming narrow bands	**2**
2	36–37 perforated scales in the lateral line; four series of scales below lateral line	*** Characidium fasciatum ***
–	34–36 perforated scales in the lateral line; two scales below lateral line	**3**
3	predorsal length less than 45% of total length; lateral vertical bars absent or without defined shape; dark maculae on caudal fin not forming defined bands	***Characidium* sp. B**
–	predorsal length up to 55% of total length; vertical bars always arranged above and below the lateral line in a “y” or “yy” shape; weak of narrow dark band on caudal fin	***Characidium* sp. C**

FAMILY CHARACIDAE

**Table:** 

1	three series of teeth in premaxillary bone	**2**
–	1 or 2 series of teeth in premaxillary bone with one or more cusps; one series of teeth in dentary	**3**
2	obtuse snout; teeth in premaxillary bone arranged in three series and two series in dentary; posterior series composed by a pair of small symphysial conic teeth on each corner	***Brycon* (*Bryconopalinus*)**
–	short and sharp snout; intermediate series of premaxillary teeth not totally separated from external one	***Piabina* (*Piabinaargentea*)**
3	pseudotimpanum present; very large scales covering preventral area	*** Phenacogaster franciscoensis ***
–	pseudotimpanum absent; preventral area with scales of small size	**4**
4	scales reaching half of the caudal fin rays	*** Knodus moenkhausii ***
–	scales just in the caudal fin base	**5**
5	adipose fin absent	**6**
–	adipose fin present	**7**
6	13–19 branched rays in anal fin; absence of rounded blotch in the median caudal fin rays	*** Hasemania nana ***
–	11–14 branched rays in anal fin; presence of rounded blotch in the base of median caudal fin rays	***Hasemania* sp.**
7	a single series of conic teeth in the premaxillary, maxillary and dentary bones; premaxilla aligned or slightly anterior to dentary in lateral view	***Oligosarcus* (*Oligosarcusargenteus*)**
–	two series of tricuspidate to multicuspidate teeth in the inner series of premaxilla; one series of tricuspidate to multicuspidate teeth in dentary	**8**
8	teeth in the inner series of premaxillary bone forming a notch	**9**
–	teeth in the inner series of premaxillary bone not forming a notch	**10**
9	absence of teeth in the maxillary bone; a conspicuous oval humeral spot arranged horizontally; hyaline fins usually yellowish, more evident in the caudal fin; more than 20 branched rays in the anal fin	*** Astyanax lacustris ***
–	presence of teeth in maxillary bone; conspicuous humeral spot vertically oriented; hyaline fins slightly reddish; iii+19 to 20 anal fin rays	***Astyanax* sp.**
10	Greater body height roughly in the middle of the pectoral fin	**11**
–	body higher at the dorsal fin origin	**12**
11	premaxilla slightly in front of dentary in lateral view; five narrow teeth aligned in the inner series of premaxillary bone; two narrow vertical lines of chromatophores surrounding border of abdominal scales; small hooks in pectoral and anal fins in mature males	***Psalidodon* sp.**
–	premaxilla aligned with dentary in lateral view; 4 or -5 wide teeth in the inner series of premaxilla (if present, the fifth teeth is too small or not aligned with others); chromatophores surrounding abdominal scales and in higher concentration on the base of the scales; developed scales in the pectoral, pelvic and anal fins	*** Psalidodon rivularis ***
12	eeth of dentary decreasing gradually in size until sixth or seventh tooth	**13**
–	teeth of dentary decreasing abruptly from the fifth tooth	**14**
13	infraorbital 3 with naked area prior to preopercle, high concentration of chromatophores; conspicuous humeral spot vertically oriented, its similar width either above and below lateral line; lateral line 38–41 (*x*¯ = 39) perforated scales in the lateral line	*** Deuterodon pedri ***
–	infraorbital 3 totally exposed, with almost no naked area prior to preopercle; infraorbital 3 shiny due to the high concentration of guanine crystals and low concentration of chromatophores; conspicuous humeral spot vertically oriented, larger above lateral line; 37 or less perforated scales in the lateral line	*** Deuterodon giton ***
14	presence of space in the symphysis of dentary; infraorbital 3 with high concentration of chromatophores	** Deuterodon aff. taeniatus **
–	absence of space in the symphysis of dentary; infraorbital 3 with low concentration of chromatophores	**15**
15	five hexacuspidate to heptacuspidate teeth in the inner series of the premaxilla; infraorbital 3 with naked area anteriorly, and below it; low concentration of chromatophores in the infraorbital 3; inconspicuous humeral spot slightly verticalized, straight anteriorly and straight or half-moon shaped posteriorly	***Deuterodon* sp.**
–	five tetracuspidate to hexacuspidate teeth in the inner series of the premaxilla; naked area anteriorly, below, and posteriorly; absence of chromatophores in the infraorbital 3; small humeral spot, no regular shaped ,sometimes slightly rounded in juveniles; no more than 1.5 scales below the lateral line; 35–37 perforated scales in the lateral line	*** Deuterodon intermedius ***

FAMILY CALLICHTHYIDAE

**Table:** 

1	coracoid bones covered by thick skin; caudal fin lobed	*** Callichthys callichthys ***
–	coracoid bones exposed; caudal fin bifurcated	*** Hoplosternum littorale ***

FAMILY TRICHOMYCTERIDAE

**Table:** 

1	absence of evident maculae, spots, streaks and/or stripes the flanks and dorsum of the body; i+7 (rarely i+8) pectoral fin rays; dark band in the median caudal fin rays	*** Trichomycterus melanopygius ***
–	body with round or rectangular spots; stripes and/or vermiculations on the flanks and/or dorsum of the body; i+6 or i+7 pectoral fin rays	**2**
2	Body spotted due to a high concentration of large maculae with no defined shape; caudal fin strongly truncated; eight branchiostegal rays; few dorsal procurrent rays (14 or 15)	***Trichomycterus* sp. A**
–	body with round or rectangular spots; stripes and/or vermiculations on flanks and/or back of the body; 7–9 branchiostegal rays; more than 20 dorsal procurrent rays	**3**
3	rounded head in dorsal view; nine branchiostegal rays; high concentration of rounded dark spots on the head and sides of the trunk, dorsum and belly, which may fuse and form small vermiculations; rounded caudal fin	***Trichomycterus* sp. B**
–	subtriangular head in dorsal view; seven branchiostegal rays; yellowish to light brown body color; rectangular or rounded sequential dark maculae at the midline of the body, sometimes fused and with a vermicular pattern, or forming a narrow stripe from the post-opercular region to the base of caudal fin; a row of rectangular sequential middorsal maculae, round or fused to maculae of the midlateral of the body; subtruncate caudal fin	*** Trichomycterus alternatus ***

FAMILY LORICARIIDAE

**Table:** 

1	depressed snout and caudal peduncle; adipose fin absent	** Loricariinae **
–	caudal peduncle not depressed; adipose fin present	**2**
2	functional spinelet of the dorsal spine; i+7 dorsal fin rays	** Hypostominae **
–	no functional spinelet of the dorsal spine; i+7 dorsal fin rays	** Neoplecostominae **


Subfamily Loricariinae

**Table:** 

1	orbit rounded; short and wide plates with poorly developed odontodes in the dorsal and ventral region of caudal peduncle	*** Harttia intermontana ***
–	inferior region of the orbit straight; compressed and narrow plates with developed odontodes in the dorsal and ventral region of caudal peduncle	***Harttia* sp.**

Subfamily Hypostominae

**Table:** 

1	black and large spots in the head and throughout the body	***Hypostomus* sp.**
–	pale small rounded spots in the whole body, including in the fins; spine of the dorsal fin slightly smaller than predorsal distance	*** Hypostomus francisci ***

Subfamily Neoplecostominae

**Table:** 

1	large eyes (until 19.7% in HL); flat body between posterior dorsal fin ray and adipose origin; flat abdomen with no plates	***Euryochus* (*Eurochusthysanos*)**
–	small (less than 19% in HL); body usually rounded; abdomen plated or not; rounded or oval inferior lip, leaving small naked area in the ventral portion of the head	**3**
3	odontodes well developed in the first ray of pectoral fins and on the sides of head in nuptial males; odontodes with normal size in no nuptial males and females	*** Pareiorhaphis ***
4	inferior lip with maxillary barbels completely adnate; elongated and pointed urogenital papilla	*** Pareiorhaphis vetula ***
–	inferior lip with free and conspicuous maxillary barbels; papilla not developed in males	**5**
5	abdomen without plates; pectoral, pelvic and anal fins with clear color; dark caudal with clear borders	***Pareiorhaphis* sp.**
–	abdomen with small plates covered by skin, from the pectoral fin to insertion of pelvic fins; fins with pale yellow and light brown spots	*** Pareiorhaphis scutula ***
–	odontodes poorly developed in the first ray of pectoral fins in mature and not nuptial males; odontodes with normal size on the lateral margin of head; abdomen with a large number of plates	*** Neoplecostomus ***
1	maxillary barbels poorly developed; premaxillary teeth and dentary with separate cusps and large concavity between them; lateral and central cusps with similar size; no developed papillae between branches of dentary; plates between dorsal and adipose fin meeting on the back of the dorsum	***Neoplecostomus* sp. A**
–	maxillary barbels developed; premaxillary teeth and dentary with close cusps; median cusp more developed than lateral one; plates between dorsal and adipose fin not meeting	***Neoplecostomus* sp. B**


FAMILY HEPTAPTERIDAE

**Table:** 

1	short adipose fin originating posteriorly to anal fin origin, in a vertical trough	*** Phenacorhamdia tenebrosa ***
–	very long adipose fin originating anteriorly to anal fin origin in a vertical trough, meeting the posterior border of the dorsal fin	***Rhamdiaquelen* group**

FAMILY PIMELODIDAE

**Table:** 

1	palatal teeth absent; humeral process, dorsoposteriorly oriented; silver body with no obvious spots	*** Pimelodus fur ***
–	Palatal teeth arranged in joined areas with each other; humeral process posteriorly oriented; rounded dark maculae with different sizes from head to caudal peduncle	*** Duopalatinus emarginatus ***

FAMILY POECILIIDAE – subfamily Poecilliinae

**Table:** 

1	modified anal fin in a short gonopodium in males (not exceeding or reaching the tip of the dorsal fin rays, in a vertical); 1–2 dark maculae in the sides of the body, anteriorly to a vertical through dorsal fin origin	*** Poecilia reticulata ***
–	long gonopodium in males (reaching and even surpassing in a vertical trough the tip of the dorsal fin rays); vertical or slightly rectangular macula at the dorsal fin rays height or slightly posterior to them	**2**
2	gonopodium with hook in the two gonopodial appendages in males positioned close to the appendages; distal half of the appendage narrower than proximal half; urogenital papilla centralized in females, positioned between anus and the base of first anal fin ray	*** Phalloceros harpagos ***
–	gonopodium with small left hook facing down or up and forward in males, situated near to gonopodial appendage base; right hook absent; urogenital papilla directed to the right side in females, positioned laterally; anal opening in contact with first anal fin ray or close to it	*** Phalloceros uai ***

FAMILY CICHLIDAE

**Table:** 

1	upper branch of first branchial arch with lobe	*** Geophagus brasiliensis ***
–	lobe absent in first branchial arch	**2**
2	XVI-XVII+9–11 dorsal fin rays; VII-VIII+9 anal fin rays	*** Australoheros mattosi ***
–	XVIII+7 dorsal fin rays; VIII+7 anal fin rays	***Australoheros* sp.**


## Discussion

The predominance of Characiformes and Siluriformes in the study area is consistent with the pattern observed among freshwater fishes in the Neotropical region (Lowe-McConnell 1999; Alves et al. 2008; Camelier and Zanata 2014).

Although taxonomic (e.g., Menezes et al. 2007; Buckup et al. 2014; Vieira et al. 2015) and ecological approaches (e.g., Castro 1999; Sabino 1999; Castro et al. 2004; Ferreira and Casatti 2006; Felipe and Súarez 2010) to study stream fishes have increased considerably in recent years, studies of taxonomy and biology of small species in a wide area of the Neotropical region are still limited. The compilation of regional records of taxonomic and ecological diversity may support conservation plans and generate data for biogeographic analyses (Winemiller et al. 2008). We found a considerable number of small and medium sized fish species arranged in populations (sensu Vazzoler 1996) that use headwaters of the upper rio Paraúna and upper rio Santo Antônio as living and developmental areas.

Almost 32% of the whole ichthyofauna from the rio das Velhas (Alves and Pompeu 2005) and ca. 56% of the ichthyofauna from the rio Santo Antônio (Vieira 2006) were recorded in this study. When compared to the species previously registered from the upper rio Santo Antônio, this percentage is even higher than that recorded by dos Santos (2015) (40 species in the present study vs. 39 in dos Santos 2015). The ichthyofaunal richness of headwaters is usually known as low and endemic (Lowe-McConnell 1999), with species that have limited ability to travel great distances (Castro 1999).

The highest species richness was registered at ribeirão das Pedras and rio Cipó, which are in lower altitude areas (Fig. 1). As observed by Castro et al. (2003) for the species from rio Parapanema basin, species richness is associated with the longitudinal gradient in the location of sampling sites. Furthermore, it is combined with the fish regional diversity plus the physical extension of the sampling environment and biogeographic patterns of ichthyofaunistic diversity. rio Cipó presented a substantial number of exclusive species from this drainage. Since it was noted the characteristic of fast-water environments nearby the mouth of the rio Paraúna and downstream of the Paraúna waterfall, we suggested that some species prefer such environments. Some of them with migratory habits (i.e., *Salminusfranciscanus* Lima & Britski, 2007 and *Pimelodusmaculatus* Lacépède, 1803) and highly appreciated in artisanal fishery in regions among municipalities of Conceição do Mato Dentro, Congonhas do Norte, Presidente Kubitschek, Santana de Pirapama, Gouveia, and Presidente Juscelino were registered.

As expected for the Southern Espinhaço mountain range, and corroborating Alves et al. (2008), several endemic and/or endangered species were found. Two of these (*Hypomasticusthayeri* and *Bryconopalinus*) are listed in Brazil as “Endangered” and “Vulnerable”, respectively (Akama et al. 2018), or “Critically Endangered”, according to the state list for endangered fish species in state of Minas Gerais (Minas Gerais 2010). Fifteen species are endemic to the studied hydrographic basins. From the total of endemic species, 11 of them come from the rio São Francisco basin (*Prochiloduscostatus*, *Leporinusmarcgravii*, *L.taeniatus*, *Psalidodonrivularis*, *Hasemanianana*, *Phenacogasterfranciscoensis*, *Duopalatinusemarginatus*, *Pimelodusfur*, *Bunocephalushartti*, *Phallocerosuai*, and *Australoherosmattosi*) and four come from the rio Doce basin (*Deuterodonpedri*, *Harttiaintermontana*, *Pareiorhaphisscutula*, and *P.vetula*). Three endemic species (*H.nana*, *P.uai*, and *P.rivularis*) from the rio São Francisco basin were found in the rio Santo Antônio basin (Table 2). These were the first records of *H.nana* and *P.rivularis* in the adjacent basin. The occurrence of *P.uai* in a different basin instead of rio São Francisco was already mentioned in the literature (Vieira et al. 2015). On the other hand, *T.alternatus* was originally described from the rio Doce basin (Reis and de Pinna 2019), and it was registered for the first time to the rio Paraúna basin. Triques and Vono (2004) extended its distribution to the rio Jequitinhonha basin and Fricke et al. (2021) summarized its distribution to the Atlantic coastal rivers in the states of Rio de Janeiro, Minas Gerais, and Espírito Santo. However, the new species records in different basins will need further investigations to elucidate the possibilities of sharing basins or even an introduction problem due to human actions. Two species (*Leporinusamblyrhynchus* and *Poeciliareticulata*) are exotic to the studied basins. However, another three registered species (*Deuterodongiton*, *D.intermedius*, and *Knodusmoenkhausii*) are also usually considered exotic for such basins, but there is no investigation into the validity of such status.

Considering our results, we reinforce the importance of headwater environment conservation, as pointed out by Drummond et al. (2005), who defined such areas as priority for fish conservation in state of Minas Gerais. Furthermore, Menezes et al. (2007) highlighted the need of studies and surveys in order to increase the knowledge about fish species which inhabit those areas and to recognize conservation priorities in aquatic environments in the Atlantic rainforest region. In addition, records for 25% of the species were based exclusively on material from scientific collections. These results support the substantial importance of zoological collections in sampling and archiving biological diversity, and also allows the development of knowledge in research facing the conservation of biodiversity (Zaher and Young 2003; Ohara et al. 2015).

The substantial number of taxonomically inaccurate identifications (ca. 30%) and potentially new species (almost 22%) recorded herein, added to the lack of data on distributional patterns reinforces the need of studies in such areas. The considerable number of potentially new species indicates the large knowledge gap in the Espinhaço mountain range. It is important to mention that the aforementioned species have been studied by different researchers and descriptions have been made, such as the currently described *H.intermontana* and *T.melanopygius*. Also, the occurrence of many large ventures in the region, such as mining and hydroelectric power plants, make such areas high priorities for biodiversity studies, to minimize the possibility of populations and species extinctions even before they are properly recognized. The increase of knowledge about such fishes may contribute to future assessments of the conservation status and the encouragement of exploratory field expeditions of remote areas, as in the case of this study. The new results shown here can provide a better understanding about biogeographic patterns and evolution of fish at the Espinhaço mountain range and adjacent basins.

## Supplementary Material

XML Treatment for Prochilodus costatus

XML Treatment for Hypomasticus mormyrops

XML Treatment for Hypomasticus thayeri

XML Treatment for Leporellus vittatus

XML Treatment for Leporinus amblyrhynchus

XML Treatment for Leporinus copelandii

XML Treatment for Leporinus marcgravii

XML Treatment for Leporinus taeniatus

XML Treatment for Megaleporinus obtusidens

XML Treatment for Characidium fasciatum

XML Treatment for Characidium

XML Treatment for Characidium

XML Treatment for Characidium

XML Treatment for Brycon opalinus

XML Treatment for Astyanax lacustris

XML Treatment for Astyanax

XML Treatment for Deuterodon giton

XML Treatment for Deuterodon intermedius

XML Treatment for Deuterodon pedri

XML Treatment for Deuterodon aff. taeniatus

XML Treatment for Deuterodon

XML Treatment for Hasemania nana

XML Treatment for Hasemania

XML Treatment for Knodus moenkhausii

XML Treatment for Oligosarcus argenteus

XML Treatment for Phenacogaster franciscoensis

XML Treatment for Piabina argentea

XML Treatment for Psalidodon rivularis

XML Treatment for Psalidodon

XML Treatment for Hoplias intermedius

XML Treatment for Bunocephalus hartii

XML Treatment for Trichomycterus alternatus

XML Treatment for Trichomycterus melanopygius

XML Treatment for Trichomycterus

XML Treatment for Trichomycterus

XML Treatment for Callichthys callichthys

XML Treatment for Hoplosternum littorale

XML Treatment for Euryochus thysanos

XML Treatment for Harttia

XML Treatment for Harttia intermontana

XML Treatment for Hypostomus

XML Treatment for Hypostomus francisci

XML Treatment for Neoplecostomus

XML Treatment for Neoplecostomus

XML Treatment for Pareiorhaphis scutula

XML Treatment for Pareiorhaphis vetula

XML Treatment for Pareiorhaphis

XML Treatment for Phenacorhamdia tenebrosa

XML Treatment for Rhamdia quelen

XML Treatment for Pimelodus fur

XML Treatment for Duopalatinus emarginatus

XML Treatment for Gymnotus carapo

XML Treatment for Eigenmannia virescens

XML Treatment for Synbranchus marmoratus

XML Treatment for Phalloceros harpagos

XML Treatment for Phalloceros uai

XML Treatment for Poecilia reticulata

XML Treatment for Australoheros mattosi

XML Treatment for Australoheros

XML Treatment for Geophagus brasiliensis

## Supplementary materials

Supplementary material 1 Summarizes voucher information and comparative material with institutional acronyms, following Sabaj (2019). Data type molecular data Explanation note Number in brackets represent the total number of specimens. https://binary.pensoft.net/file/574539Sérgio Alexandre dos Santos, Marcelo Ribeiro de Britto
