# Supplementary material for: The ichthyofauna of a poorly known area in the middle-southern Espinhaço mountain range, state of Minas Gerais, Brazil: diagnostics and identification keys
Source: Zookeys. 2021 Aug 3;1054:25–66. doi: 10.3897/zookeys.1054.67554 (PMC8355004; doi:10.3897/zookeys.1054.67554)
Supplement: Supplementary material 1 — Summarizes voucher information and comparative material with institutional acronyms, following Sabaj (2019). [file zookeys-1054-025-s001.pdf]

Supplementary file 1

Supplementary file 1 summarizes voucher information and comparative material with institutional acronyms, following Sabaj (2019). Number in brackets represent the total number of specimens.

Parodontidae: *Apareiodon ibitiensis* Amaral Campos, 1944 ZUEC 7618 (3); **Curimatidae:** *Cyphocharax gilbert* (Quoy & Gaimard, 1824) MZUSP 54699 (1); **Prochilodontidae:** *Prochilodus costatus* Valenciennes, 1850 MZUSP 73822 (1); **Anostomidae:** *Hypomasticus mormyrops* (Steindachner, 1875), MNRJ 47811 (1), MNRJ 47854 (1), MNRJ 47843 (6); *Hypomasticus thayeri* Borodin, 1929 ZUEC 7606 (22), MNRJ 47795 (2); *Leporellus vittatus* (Valenciennes, 1850) MZUSP 73652 (1); *Leporinus amblyrhynchus* Garavello & Britski, 1987 MZUSP 73653 (1); *Leporinus copelandii* (Steindachner, 1875) MNRJ 46910 (1); *Leporinus marcgravii* Lütken, 1875 MZUSP 73657 (1); *Leporinus taeniatus* Lütken, 1875 MZUSP 73663 (2); *Megaleporinus obtusidens* (Valenciennes, 1837) MZUSP 73671 (1); **Crenuchidae:** *Characidium fasciatum* Reinhardt, 1867 NMW (2, syntypes), ZUEC 7632 (2), ZUEC PIS 7014 (8), ZUEC 6389 (5), ZUEC 7285 (2), MNRJ 48435 (5); *Characidium zebra* Eigenmann, 1909 NMW 69263(1, paratype); *Characidium* sp. A MNRJ 47844 (4), MNRJ 22405 (18), ZUEC 7601 (7), MNRJ 47802 (3), MNRJ 47816 (6), MNRJ 47817 (2), MNRJ 47828 (4), MNRJ 47844 (4), MNRJ 47882 (1), MNRJ 47894 (8), MNRJ 46861 (24), MNRJ 46832 (10); *Characidium* sp. B MNRJ 47868 (1), MNRJ 46911 (21); *Characidium* sp. C MCNIP 0976 (2), MCNIP 1026 (5), ZUEC 7604 (1), MNRJ 47788 (2), MNRJ 47797 (2), MNRJ 47813 (4), MNRJ 47856 (1), MNRJ 47877 (1), MNRJ 48460 (5), MNRJ 47801 (1), MNRJ 47827 (1), MNRJ 47895 (1); **Bryconidae:** *Brycon opalinus* Cuvier, 1819 MNRJ 47796 (4), MNRJ 47812 (2), MNRJ 47837 (1), MNRJ 47855 (1), MNRJ 47890 (1); **Characidae:** *Phenacogaster franciscoensis* Eigenmann, 1911 MZUSP 73661 (1), *Astyanax lacustris* (Lütken, 1875) NMW 57540 (1, syntype), ZUEC PIS 7284 (2), MZUSP 73657 (1), MNRJ 46885 (4), MNRJ 46896 (5), MNRJ 46905 (13), MNRJ 46916 (19), MNRJ 46921 (3), MNRJ 48409 (5), MNRJ 48415 (8), MNRJ 48482 (1), MNRJ 48488 (2), MNRJ 48449 (71), MNRJ 48498 (7), MNRJ 48505 (6), MNRJ 46850 (14), MNRJ 45836 (1), MNRJ 45838 (1), MNRJ 45844 (11), MNRJ 46900 (8), MNRJ 45858 (1), MNRJ 48508 (5), MNRJ 48521 (10), MNRJ 48529 (1), MNRJ 46892 (5), MNRJ 48427 (3), MNRJ 48433 (13); *Astyanax* sp. MNRJ 48130 (1), MNRJ 48375 (4), MNRJ 48509 (1), MNRJ 48512 (2), MNRJ 48132 (2); *Deuterodon* sp. MCP 44974 (8); *Deuterodon giton* (Eigenmann, 1908) MCP 48270 (1), MCP 44409 (1), MCP 44411 (6), MZUSP 104717 (15), MNRJ 47863 (1), MNRJ 48129 (2), MNRJ 47792 (2), MNRJ 48401 (5), MNRJ 47786 (3), MNRJ 47851 (5); *Deuterodon intermedius* (Eigenmann, 1908) MCP 15408 (2), MZUSP

112746 (15), MZUSP 073150 (10), MNRJ 47839 (14), MNRJ 47866 (10), MNRJ 47879 (29), MNRJ 47850 (181), MNRJ 47886 (60), MNRJ 47825 (2), MNRJ 47872 (76), MNRJ 47880 (12), MNRJ 47798 (3), MNRJ 50233 (6), MNRJ 47893 (6), MNRJ 47791 (4), MNRJ 48290 (6), MNRJ 47840 (12), MNRJ 47873 (1); *Deuterodon pedri* Eigenmann, 1908 MCNIP 0990 (4), MCP 47661 (5), MNRJ 47835 (1), MNRJ 47852 (5), MNRJ 47864 (1), MNRJ 47887 (9), MNRJ 47874 (4), MNRJ 47793 (9), MNRJ 47809 (4), MNRJ 46906 (9), MNRJ 50232 (7), MNRJ 48291 (11), MNRJ 47874 (4), MNRJ 48381 (41), MNRJ 48373 (9); *Deuterodon* aff. *taeniatus* MNRJ 47808 (32), MNRJ 47862 (45), MNRJ 45824 (97), MNRJ 47790 (55), MNRJ 47785 (18), MNRJ 47834 (23), MNRJ 45813 (22), MNRJ 46917 (27), MNRJ 46852 (10), MNRJ 48374 (1), MNRJ 48392 (5), MNRJ 46922 (7), MNRJ 48391 (2), MNRJ 45852 (51); *Psalidodon* sp. MNRJ 48410 (19), MNRJ 48128 (43); *Psalidodon fasciatus* (Cuvier, 1819) MZUSP 18929 (9), ZUEC 7289 (8); *Psalidodon rivularis* (Lütken, 1875) NMW 57707 (5, syntypes), MCNIP 0988 (1), MCP 49309 (6), MCP 44905 (15), MCP 34211 (36), MZUSP 28138 (9), MZUSP 28440 (5), MZUSP 28140 (4), MZUSP 18928 (20), MNRJ 9205 (4), ZUEC 6900 (14), ZUEC 6988 (17), MNRJ 46897 (27), MNRJ 48483 (9), MNRJ 48489 (17), MNRJ 48450 (72), MNRJ 48499 (7), MNRJ 48131 (8), MNRJ 46851 (5), MNRJ 46860 (2), MNRJ 45831 (100), MNRJ 45839 (10), MNRJ 45856 (2), MNRJ 45859 (20), MNRJ 48516 (123), MNRJ 48522 (31), MNRJ 48524 (4), MNRJ 48531 (3), MNRJ 46893 (164), MNRJ 48428 (14), MNRJ 48434 (11); *Hasemania nana* (Lütken, 1875) ZUEC 7288 (11), ZUEC 7624 (16), MZUSP 75320 (9), MZUSP 75328 (5), MZUSP 39342 (1), MZUSP 39196 (4), MZUSP 39426 (2), MZUSP 39171 (23), MZUSP 39536 (12), MZUSP 39308 (8), MZUSP 39514 (1), MZUSP 39547 (1), MZUSP 18926 (1), MZUSP 37173 (9), MNRJ 43581 (61), MNRJ 31857 (3), MNRJ 48451 (15), MNRJ 47799 (1), MNRJ 47841 (1), MNRJ 47875 (1), MNRJ 47888 (1), MNRJ 47896 (1), MNRJ 46907 (6), MNRJ 46918 (5), MNRJ 48473 (2), MNRJ 48440 (62), MNRJ 48458 (14), MNRJ 48484 (20), MNRJ 48500 (26), ZUEC 6948 (6), ZUEC 6954 (7); *Hasemania* sp. ZUEC 7272 (1), MNRJ 45840 (3), MNRJ 45845 (7), MNRJ 46887 (21), MNRJ 46902 (5), MNRJ 48416 (3), MNRJ 48422 (1), MNRJ 48510 (2), MNRJ 48513 (2), MNRJ 48523 (20), MNRJ 48525 (19); *Knodus moenkhausii* (Eigenmann & Kennedy, 1903) MNRJ 47800 (10), MNRJ 47826 (2), MNRJ 47842 (2), MNRJ 47867 (6), MNRJ 47881 (2), MNRJ 46908 (115), MNRJ 46919 (5), MNRJ 46923 (8), MNRJ 48474 (314), MNRJ 48441 (152), MNRJ 48459 (39), MNRJ 48466 (7); *Oligosarcus argenteus* Günther, 1864 MNRJ 47787 (9), MNRJ 47794 (12), MNRJ 47810 (6), MNRJ 47836 (4), MNRJ 47853 (12), MNRJ 47865 (2), MNRJ 47876 (1), MNRJ 47889 (3), MNRJ 45853 (29), MNRJ 45814 (16), MNRJ 45825 (10), MNRJ 46909 (2), MNRJ 46920 (3), MNRJ 46924 (20), MNRJ 48467 (1), MNRJ 48517 (3), MNRJ 48393 (18), MNRJ 48383 (2), MNRJ 48376 (1); *Piabina argentea* Reinhardt, 1867 MZUSP 110200 (8); *Serrapinnus heterodon* (Eigenmann, 1915) MZUSP 50999(2);

Erythrinidae: *Hoplias intermedius* (Günther, 1864) MZUSP 54696 (2), MZUSP 73655 (1), ZUEC 7286 (3), ZUEC 7652 (1), ZUEC 4238 (1), ZUEC 4239 (1), MNRJ 45823 (2), MNRJ 48526 (1), MNRJ 45841 (3), MNRJ 47803 (1), MNRJ 47814 (1), MNRJ 47878 (3), MNRJ 48394 (2), MNRJ 46903 (2), MNRJ 48417 (1), MNRJ 46888 (1), MNRJ 48384 (2), MNRJ 48514 (1), MNRJ 46863 (2), MNRJ 48533 (1), MNRJ 48429 (3), MNRJ 46853 (8), MNRJ 48518 (2), MNRJ 45832 (4), MNRJ 46894 (1), MNRJ 46898 (1), MNRJ 48436 (2), MNRJ 48485 (1), MNRJ 48411 (1), MNRJ 48452 (1), MNRJ 46912 (2); **Aspredinidae:** *Bunocephalus hartti* Carvalho, Cardoso, Friel & Reis, 2015 MZUSP 64227 (3, paratypes), MZUSP 72745 (1); **Trichomycteridae:** *Cambeva variegata* (Costa, 1992) MNRJ 31571 (60); *Trichomycterus alternatus* (Eigenmann, 1917) MCP 31878 (1), MNRJ 47789 (2), MNRJ 47807 (1), MNRJ 47815 (5), MNRJ 47823 (6), MNRJ 47838 (2), MNRJ 47857 (1), MNRJ 47869 (1), MNRJ 47884 (2), MNRJ 47891 (11), MNRJ 47899 (3), MNRJ 45815 (49), MNRJ 45817 (4), MNRJ 45826 (9), MNRJ 46889 (3), MNRJ 46925 (5), MNRJ 48412 (12), MNRJ 48418 (38), MNRJ 48425 (65), MNRJ 48430 (5), MNRJ 48437 (2), MNRJ 48476 (31), MNRJ 48443 (10), MNRJ 48461 (70), MNRJ 48470 (6), MNRJ 48536 (1), MNRJ 48403 (113), MNRJ 48405 (1), MNRJ 48395 (15), MNRJ 48400 (1), MNRJ 48385 (7), MNRJ 48386 (2), MNRJ 48377 (35), MZUSP 75254 (3); *Trichomycterus auroguttatus* Costa, 1992 MZUSP 43341 (holotype), MZUSP 43342 (4, paratypes), MZUSP 87832 (1); *Trichomycterus brasiliensis* Lütken, 1874 MNRJ 983 (11), MZUSP 28139 (2), ZUEC 157-159 (3), ZUEC 160-169 (8), ZUEC PIS 3730 (3); *Trichomycterus caudofasciatus* Alencar & Costa, 2004 MNRJ 22396 (21); *Trichomycterus immaculatus* (Eigenmann & Eigenmann, 1889) MNRJ 44073 (5), MNRJ 18396 (2), MNRJ 22453 (6); *Trichomycterus itacambirussu* Triques & Vono, 2004 MNRJ 32122 (1); *Trichomycterus jequitinhonhae* Triques & Vono, 2004 MNRJ 22481 (3); *Trichomycterus melanopygius* Reis, dos Santos, Britto, Volpi & de Pinna, 2020 MNRJ 46926 (5, paratypes), MNRJ 46933 (2, paratypes), MNRJ 48462 (5, paratypes), MNRJ 48469 (1, paratype), MNRJ 47902 (1), MNRJ 45816 (1), MNRJ 45827 (3), MNRJ 46854 (1), MNRJ 46865 (1), MNRJ 48477 (1), MNRJ 48404 (1); *Trichomycterus novalimensis* Barbosa & Costa, 2010 MZUSP 114034 (26), MCP 3816 (1); *Trichomycterus pauciradiatus* Alencar & Costa, 2006 MNRJ 31658 (7); *Trichomycterus pradensis* Sarmento-Soares, Martins-Pinheiro, Aranda & Chamon, 2005 MNRJ 28488 (4, paratypes), MNRJ 28490 (6, paratypes), MNRJ 32050 (27), MNRJ 18045 (6); *Trichomycterus reinhardti* (Eigenmann, 1917) MCP 34202 (2), MCP 3816 (1), MNRJ 21413 (6); *Trichomycterus* sp. A MNRJ 47824 (2), MNRJ 47829 (1), MNRJ 47830 (3), MNRJ 47845 (4), MNRJ 47885 (3), MNRJ 47901 (3); *Trichomycterus* sp. B MNRJ 47900 (1), MNRJ 46864 (1), MNRJ 46932 (1), MNRJ 48478 (1); **Callichthyidae:** *Callichthys callichthys* (Linnaeus, 1758) MCNIP 0854 (3), MCNIP 0856 (1), MCNIP 0983 (1), MNRJ 45818 (2), MNRJ 48475 (2), MNRJ 48442 (3), MNRJ 48468 (1), MNRJ 48501 (6),

MNRJ 48402 (1); *Hoplosternum littorale* (Hancock, 1828) Uncatalogued; **Loricariidae:** *Euryochus thysanos* Pereira & Reis, 2017 MNRJ 47870 (1), MNRJ47805 (2), MNRJ 47832 (1), MNRJ 47847 (1), MNRJ 47819 (2), MNRJ 47897 (3); *Neoplecostomus doceensis* Roxo, Silva, Zawadzki & Oliveira, 2014 MZUSP 107368 (2, paratypes), MCNIP 421 (3), MNRJ 42254 (1), MNRJ 22457 (23), MNRJ 22458 (9), ZUEC PIS 8160 (2); *Neoplecostomus franciscoensis* Langeani, 1990 MZUSP 38577 (holotype), MCP 42428 (6), MCP 34203 (2); *Neoplecostomus paranensis* Langeani, 1990 MNRJ 31630 (73); *Neoplecostomus* sp. A MCNIP 992 (2), MCNIP 1030 (2), MNRJ 47806 (4), MNRJ 47820 (9), MNRJ 47833 (2), MNRJ 47848 (4), MNRJ 47883 (4), MNRJ 47898 (2), MNRJ 46866 (2), MNRJ 46928 (1), MNRJ 46935 (1), MNRJ 48534 (3); *Neoplecostomus* sp. B MNRJ 48423 (7), MNRJ 48431 (5), MNRJ 48438 (1); *Harttia carvalhoi* Miranda Ribeiro, 1939 MNRJ 24019 (47); *Harttia gracilis* Oyakawa, 1993 MNRJ 23981 (4); *Harttia intermontana* Oliveira & Oyakawa, 2019 MNRJ 38458 (1); *Harttia leiopleura* Oyakawa, 1993 MZUSP 43265 (2, paratypes); *Harttia longipinna* Langeani, Oyakawa & Montoya-Burgos, 2001 MNRJ 21570 (1); *Harttia loricariformis* Steindachner, 1877 NMW 46346 (1, lectotype), NMW 46344 (1, paralectotype), NMW 46345 (2, paralectotypes); *Harttia novalimensis* Oyakawa, 1993 MZUSP 43263 (3, paratypes), MNRJ 38492 (3); *Harttia torrenticola* Oyakawa, 1993 MZUSP 43286 (2, paratypes), MNRJ 21557 (4), MNRJ 32720 (15), MNRJ 29000 (8), MNRJ 31762 (52); *Harttia* cf. *gracilis* MNRJ 31739 (2), MNRJ 26514 (2); *Harttia* cf. *longipinna* ZUEC 7619 (3), ZUEC 7633 (10); *Harttia* sp. MNRJ 47804 (2), MNRJ 47818 (3); *Hypostomus francisci* (Lütken, 1874) MZUSP 37162 (4); *Hypostomus* sp. MNRJ 47846 (1); *Pareiorhaphis mutuca* (Oliveira & Oyakawa, 1999) MCP 46305 (4); *Pareiorhaphis nasuta* Pereira, Vieira & Reis, 2007 MCP 41764 (holotype), MCP 38808 (2, paratypes); *Pareiorhaphis scutula* Pereira, Vieira & Reis, 2010 MCP 46306 (3), MCP 46307 (5), MCP 48755 (1), MNRJ 42140 (3), MNRJ 42253 (6), MNRJ 38452 (5), ZUEC 6914 (15), ZUEC 6897 (5), ZUEC 6995 (7), ZUEC 6890 (3), MCP 42388 (6), MNRJ 47821 (1), MNRJ 46867 (5), MNRJ 46927 (1), MNRJ 46929 (6), MNRJ 48464 (17), MNRJ 48471 (14), MNRJ 48535 (10), MNRJ 48387 (2), MNRJ 48378 (4); *Pareiorhaphis vetula* Pereira, Lehmann & Reis, 2016 MCP 49693 (holotype), MCP 47773 (2), MNRJ 46936 (11), MNRJ 48465 (7), MNRJ 48406 (1), MNRJ 48396 (2) MNRJ 48390 (1); *Pareiorhaphis* sp. MNRJ 48424 (3); **Heptapteridae:** *Phenacorhamdia tenebrosa* (Schubart, 1964) MZUSP 73653 (1); *Rhamdia quelen* group MZUSP 73659 (1), MNRJ 47822 (1), MNRJ 45819 (2), MNRJ 46855 (1), MNRJ 45846 (1), MNRJ 48490 (1); **Pimelodidae:** *Duopalatinus emarginatus* (Valenciennes, 1840) MZUSP 73819 (1); *Pimelodus fur* (Lütken, 1864) MZUSP 73821 (1); **Gymnotidae:** *Gymnotus carapo* group MNRJ 47849 (1), MNRJ 47858 (1), MNRJ 45820 (8), MNRJ 45828 (11), MNRJ 46856 (8), MNRJ 46857 (1), MNRJ 45833 (5), MNRJ 45834 (1), MNRJ 45842 (1), MNRJ 45847 (7), MNRJ 45848 (1), MNRJ 46890 (1), MNRJ

45860 (1), MNRJ 46930 (2), MNRJ 48413 (5), MNRJ 48419 (3), MNRJ 48479 (1), MNRJ 48444 (10), MNRJ 48486 (2), MNRJ 48453 (2), MNRJ 48502 (2), MNRJ 48511 (1), MNRJ 48519 (4), MNRJ 48527 (1), MNRJ 48407 (2), MNRJ 48397 (3); **Sternopygidae:** *Eigenmannia virescens* (Valenciennes, 1836) MZUSP 73660 (1); **Poeciliidae:** *Phalloceros harpagos* Lucinda, 2008 MNRJ 46858 (253), MNRJ 46868 (4), MNRJ 48537 (12); *Phalloceros uai* Lucinda, 2008 MNRJ 45854 (19), MNRJ 45821 (123), MNRJ 45829 (96), MNRJ 46913 (3), MNRJ 48480 (174), MNRJ 48446 (95), MNRJ 48408 (138), MNRJ 48398 (52), MNRJ 48389 (9), MNRJ 48380 (10); *Poecilia reticulata* Peters, 1859 MNRJ 45857 (3), MNRJ 46891 (6), MNRJ 46895 (3), MNRJ 46899 (1), MNRJ 46904 (21), MNRJ 46914 (5), MNRJ 48414 (10), MNRJ 48420 (11), MNRJ 48426 (1), MNRJ 48432 (21), MNRJ 48439 (4), MNRJ 48447 (74), MNRJ 48487 (11), MNRJ 48504 (1), MNRJ 48506 (46), MNRJ 48515 (16); **Synbranchidae:** *Synbranchus marmoratus* group MNRJ 48448 (2); **Cichlidae:** *Australoheros mattosi* Ottoni, 2012 MNRJ 48454 (7); *Australoheros* sp. MNRJ 46859 (1); *Geophagus brasiliensis* (Quoy & Gaimard, 1824) MNRJ 46931 (5), MNRJ 45822 (6), MNRJ 45830 (16), MNRJ 46915 (2), MNRJ 48481 (1), MNRJ 48445 (8), MNRJ 48455 (27), MNRJ 48503 (5), MNRJ 48507 (3), MNRJ 48399 (4), MNRJ 48388 (1), MNRJ 48379 (1), MNRJ 47871 (1), MNRJ 45855 (1), MNRJ 45835 (3), MNRJ 45837 (10), MNRJ 458437 (5), MNRJ 48520 (3), MNRJ 48528 (3), MNRJ 48530 (1).
